# Transcriptome Profiling of the Elongating Internode of Cotton (*Gossypium hirsutum* L.) Seedlings in Response to Mepiquat Chloride

**DOI:** 10.3389/fpls.2019.01751

**Published:** 2020-01-28

**Authors:** Li Wang, Ying Yin, Li-Feng Wang, Menglei Wang, Miao Zhao, Ye Tian, Yong-Fang Li

**Affiliations:** College of Life Sciences, Henan Normal University, Xinxiang, China

**Keywords:** mepiquat chloride, cotton seedlings, internode, phytohormone, secondary metabolism, transcription factors, cell division, cell expansion

## Abstract

The plant growth retardant mepiquat chloride (MC) has been extensively used to produce compact plant canopies and increase yield in cotton (*Gossypium hirsutum* L.). Previous studies showed that MC reduced plant height and internode length by inhibiting GA biosynthesis and cell elongation. However, whether there are other molecular mechanisms underlying MC-induced growth retardation has remained largely unknown. In the present study, we conducted histological, transcriptomic, and phytohormone analyses of the second elongating internodes of cotton seedlings treated with MC. Histological analysis revealed that the MC shortened the internodes through suppressing both cell division and cell elongation. Consistent with the observed phenotype, many genes related to cell growth were significantly downregulated by MC. Transcriptome profiling showed that the expression of genes related not only to GA, but also to auxin, brassinosteroid (BR), and ethylene metabolism and signaling was remarkably suppressed, whereas that of genes related to cytokinin (CK) and abscisic acid (ABA) metabolism was induced by MC. Consistent with the expression pattern, significant decrease of endogenous GA, auxin, and BR content, but an increase in CK content was observed after MC treatment. Most of these hormone related genes displayed opposite regulation pattern by exogenous GA_3_ treatment compared to MC; simultaneous application of MC and GA_3_ could alleviate the genes expression changes induced by MC treatment, indicating MC does not directly affect other plant hormones, but through the inhibition of the GA biosynthesis. In addition, the expression of genes related to secondary metabolism and many transcription factors (TFs) were differentially regulated by MC. In summary, we confirmed the important role of GA in MC-induced growth inhibition of cotton, and further found that other hormones were also involved in this process in a GA-dependent manner. This study provides novel insights into the molecular mechanism underlying the MC-mediated inhibition of internode elongation in cotton seedlings.

## Introduction

Cotton (*Gossypium hirsutum* L.) is an important economic crop; it is widely cultivated worldwide for its natural fiber and oilseed. As a perennial shrub, cotton plants exhibit indeterminate growth. Under favorable growing conditions, excessive vegetative growth often occurs, leading to serious production problems such as auto-shading, fruit abortion, delayed maturity, and yield reduction (Zhao and Oosterhuis, 2000). Plant growth retardants are commonly used to inhibit excessive vegetative growth and control plant shape.

 Gibberellin (GA) is a key kind of hormones that regulate stem and internodal elongation by affecting cell division and expansion (Wang et al., 2018). GAs promote plant growth by stimulating the degradation of DELLA proteins—the major repressors of GA signaling—by binding to the receptor—GIBBERELLIN INSENSITIVE DWARF1 (GID1)—through the ubiquitin–proteasome pathway (Velde et al., 2017). When the content of active GAs decreases, DELLA proteins accumulate and interact with transcription factors (TFs) to suppress the expression of their target genes and further suppress cell division or cell elongation (Fukazawa et al., 2017; Velde et al., 2017).

Plant growth retardants can interfere with the endogenous content of not only GAs but also other plant hormones (Rademacher, 2000). Paclobutrazol (PAC), uniconazole, and prohexadione-Ca (Pro-Ca) are well-known anti-GA plant growth retardants that are widely applied in various plants. PAC could reduce GA content, but increased cytokinin (CK) and abscisic acid (ABA) content in the buds and leaves of mango (Upreti et al., 2013). Uniconazole and Pro-Ca application yielded similar results in duckweed (*Landoltia punctata*) (Liu et al., 2015) and in the shoots of wheat and oilseed rape, respectively (Grossmann et al., 1994). Uniconazole has been reported to inhibit brassinosteroid (BR) biosynthesis in *Pisum sativum* (Yokota et al., 1991). PAC and uniconazole could reduce ethylene levels in soybean and wheat seedlings (Kraus et al., 1992) and in pineapple (Min and Bartholomew, 1996), respectively. Recent studies have shown that plant growth retardants could affect the expression of hormone-related genes. PAC affected the expression of many hormone-related genes in *Jatropha* (Seesangboon et al., 2018) and *Agapanthus praecox* (Zhang et al., 2015). The expression of genes related to GA, CK, and ABA was altered by uniconazole in duckweed (Liu et al., 2015). However, whether the biosynthesis and signaling of these hormones are directly affected by plant growth retardants or not is unknown. Apart from plant hormones, many other factors can also affect plant architecture, such as cell wall-related genes (Marowa et al., 2016; Minami et al., 2018), genes involved in secondary metabolism (Dare et al., 2013; Muro-Villanueva et al., 2019), and TFs (Davière et al., 2014; Kuijt et al., 2014). In plants, cell expansion is mainly mediated by the selective loosening of cell walls (Wolf, 2017). Many cell wall-related plant mutants show abnormal growth and morphogenesis (Pien et al., 2001). Flavonoids and lignin are important secondary metabolites during plant growth and development. Flavonoids are required for internode elongation and leaf expansion in apple (Dare et al., 2013), fiber elongation in cotton (Tan et al., 2013), and pollen tube growth and development in maize and tomato (Pollak et al., 1995; Schijlen et al., 2007). Lignin is a major structural component of the secondary cell wall, providing mechanical strength and hydrophobicity to the vascular system, especially in the stem (Muro-Villanueva et al., 2019). Modification of its metabolism can directly affect plant growth. Lignin modification-induced dwarfism has been reported in Arabidopsis, poplar, tobacco, and alfalfa (Pinçon et al., 2001; Leplé et al., 2007; Shadle et al., 2007). TFs are important regulators of plant growth and development; they activate or inhibit the transcription of downstream genes in response to environmental stimuli. Multiple TFs have been reported to alter plant architecture by regulating cell proliferation and/or expansion. For example, the members of the TCP family are typically associated with cell proliferation and expansion (Martín-Trillo and Cubas, 2010). Growth-regulating factors (GRFs) positively regulate leaf size and stem elongation by promoting cell proliferation or expansion (Kuijt et al., 2014; Jiang et al., 2018). In addition, TFs play key roles in integrating signal transduction of different phytohormones (Zhiponova et al., 2014; Davière and Achard, 2016).

GA biosynthesis inhibitors include onium compounds, nitrogen-containing heterocyclic compounds, structural mimics of 2-oxoglutaric acid, and acylcyclohex-anediones (Rademacher, 2000). Mepiquat chloride (MC) belongs to onium compounds (Rademacher, 2000). Cotton is more sensitive to MC compared with other plant species such as wheat, maize, and soybean for unknown reason (Rademacher, 2000); therefore, MC is commonly used in cotton to control growth, maximize yield, and improve fiber quality by reducing leaf area and shortening internodes (Reddy et al., 1990; Rademacher, 2000; Siebert and Stewart, 2006; Ren et al., 2013). Because of its chemical structure similarity to chlorocholine chloride (CCC), the regulatory mechanism of which has been relatively well studied in plants, MC is hypothesized to function by specifically inhibiting the activity of copalyl diphosphate synthase (CPS) in the early steps of GA biosynthesis (Shechter and West, 1969; Rademacher, 2000). Previously, we showed that MC decreased the expression of genes related to GA metabolism and signaling pathway, reduced GA contents, and inhibited cell elongation in the internodes of cotton seedlings grown in soil (Wang et al., 2014), however, the whole transcriptional regulatory network underlying MC-mediated growth inhibition remains largely unknown. Whether other hormones are affected by MC is unknown in cotton. To better disclose the signal pathways and genes associated with the growth inhibition of MC, global transcriptome profiling of the elongating internodes of cotton seedlings in response to MC was conducted using hydroponically grown cotton seedlings, together with phenotype, cell morphology, and phytohormone analyses. This study is useful for deep understanding the molecular mechanism underlying MC-mediated growth inhibition in cotton, it may also be helpful to disclose the effect of other GA biosynthesis inhibitors in plants. In addition, a better understanding the molecular mechanism of MC action may help accelerate the molecular breeding process of cotton.

## Materials and Methods

### Plant Materials and Treatments

*Gossypium hirsutum* (CCRI49) seeds were obtained from Institute of Cotton Research of Chinese Academy of Agricultural Sciences (Anyang, China). Cotton seeds were immersed in water for 8 h at 37°C, and then germinated in sand at 28°C in the dark for 3 days. Subsequently, uniform seedlings were transferred to plastic pots filled with aerated half-strength Hoagland solution and grown hydroponically in a growth chamber with a 14 h photoperiod at a 28/20°C day/night temperature cycle, with a light intensity of 550 µmol m^−2^ s^−1^.

Based on the findings of our previous study (Wang et al., 2014), we used 80 mg/L of MC for treatment in this study. The MC standard (purity, 97.0%) was supplied by Hebei Guoxin ahadzi-nonon Biological Technology Co., Ltd. (Hejian, Hebei, China). At the three-leaf stage, 120 seedlings were randomly divided into two groups: MC (90 seedlings) and deionized water as control (30 seedlings). Both treatments were applied to the seedlings by foliar spray. The experiment was performed with three independent biological replicates. The length of each internode was measured at 10 days after treatment. The length of the second internode, an elongating internode, was measured before plants were subjected to MC treatment, and then at 2-day intervals for 10 days. The second internodes were harvested at 0, 48, 72, and 96 h after treatment for RNA extraction and transcriptome analysis. The upper halves of the second internodes were sampled at 10 days after treatment for endogenous hormone content and cell morphology analyses.

For simultaneous treatment of MC and exogenous GA_3_, 160 seedlings at the three-leaf stage were randomly divided into four groups. The four groups were treated by foliar spraying of deionized water (control), 80 mg/L MC, 100 µM GA_3_ (Sigma-Aldrich), and combination of 80 mg/L MC and 100 µM GA_3_. The second internodes were harvested at 72 h after treatment for RNA extraction and quantitative real-time PCR analysis. The experiment was performed with three independent biological replicates.

### Histological Analysis

Ten second elongating internodes were collected for the measurement of cell length and cell number of MC-treated and control plants 10 days after MC treatment. The upper zones of the internodes (approximately 5 mm each) were fixed in formalin–acetic acid–alcohol fluid containing 5% acetic acid, 45% ethanol, and 5% formaldehyde. The samples were dehydrated in a graded ethanol/tert-butanol series, embedded in paraffin, and sectioned longitudinally to 10 µm on a rotary microtome (Leica Instruments GmbH; Wetzlar, Germany). Paraffin sections were stained with fast green and digitized using Pannoramic P250 Flash (3DHistech; Hungary). The length of about 100 cells per internode was measured from the longitudinal sections of cortex cells by using Caseviewer software 3.3 (3D HISTECH Ltd., Budapest, Hungary). The cell number in each internode was estimated as the ratio of internode length to mean cell length.

### RNA Extraction and Transcriptome Analysis

Total RNA was extracted from the second internodes by using RNAprep Pure Plant Kit (Tiangen, Beijing, China) and then treated with RNase-free DNase I (Takara) to remove genomic DNA. The RNA was electrophoresed on 1% agarose gels for monitoring RNA degradation and contamination. RNA purity, concentration, and integrity were further assessed using Nanodrop (Thermo Scientific, USA) and Agilent 2100 (Agilent Technologies, CA, USA). Twelve RNA-Seq libraries (4 time points × 3 independent biological replicates) were prepared using the Next Ultra Directional RNA Library Prep Kit (NEB), following manufacturer's instructions. In brief, mRNA was purified from total RNA by using poly-T oligo-attached magnetic beads. The mRNA was fragmented into small pieces by using a divalent cation under elevated temperature, and then first-strand cDNA was synthesized by using random hexamer primers and M-MuLV Reverse Transcriptase (NEB). Following cDNA synthesis, the second-strand cDNA was synthesized using DNA polymerase I and RNaseH. Subsequently, the cDNA fragments were adenylated and ligated with adaptors. The ligation products were purified and then enriched using PCR amplification to create libraries. RNA-Seq libraries were sequenced using an Illumina HiSeq4000 platform at Novogene Bioinformatics Institute (Beijing, China).

### Transcriptome Sequence Processing and Analysis

Raw sequencing data in FASTQ format were first filtered to remove adapter sequences and low-quality reads (reads with ambiguous bases, > 10% and more than 50% bases with Q ≤ 20) to obtain clean reads by using custom Perl scripts. The clean reads were mapped to the cotton reference genome (https://www.cottongen.org/species/Gossypium_hirsutum/nbi-AD1_genome) by using HISAT software program (Kim et al., 2015). Gene annotation was performed by BLASTX search against Arabidopsis protein database version 10 (http://www.arabidopsis.org) with a threshold of 10^−5^. Gene expression level was calculated using HTSeq v0.5.4p3 and normalized to the FPKM value (Anders and Huber, 2010). The DESeq package (ver. 2.1.0) was used for pairwise gene expression comparisons (Anders and Huber, 2010). The DEGs (48 vs. 0 h, 72 vs. 0 h, and 96 vs. 0 h) were identified using a FDR of ≤ 0.05, absolute value of log_2_ (fold change) ≥ 1; and more than 1 FPKM in at least one sample of each comparison. Principal component analysis (PCA) was performed using the R statistical environment (version3.1.1).

### GO and KEGG Enrichment Analysis

GO enrichment analysis of DEGs was performed using the GOseq R package (Young et al., 2010), with corrected *P*-value < 0.05 regarded as significant. KEGG pathways analysis of the DEGs was performed using KOBAS software (Xie et al., 2011).

### Quantitative Real-Time PCR Analysis

The same total RNA samples used for transcriptome analysis were used for quantitative real-time PCR (qPCR) analysis. DNA-free total RNA (1 μg) was reverse transcribed into cDNA by using oligo (dT)20 primers and MMLV reverse transcriptase (Takara, Japan). The cDNAs were diluted 10-fold for qPCR analysis. Real-time PCR mixture (20 μL in total volume) included 10 μL of SYBR Premier Ex TaqII mix (Takara, Japan), 0.5 μL of each primer (10 μM), 2 μL of diluted cDNA, and 7 μL DNase-free water. qPCR was performed on a LightCycler 96 real-time PCR instrument (Roche, Switzerland), initiated at 95°C for 10 min, followed by 45 cycles at 95°C for 10 s and 60°C for 30 s. Melting curve analysis was performed at 95°C for 10 s, 65°C for 60 s, and 97°C for 1 s. All reactions were performed in three independent biological replicates with two technical replicates each. *GhActin4* was used as an internal control to normalize the gene expression. Primers used for qRT-PCR (**Table S1**) were designed using Primer3 software (http://primer3.sourceforge.net).

### Hormone Quantification

At 6 days after MC treatment, the second internode was used for the measurement of the content of endogenous GA_3_, GA_4_, IAA, trans-zeatin, and brassinolide. Endogenous GA_3_ and GA_4_ were extracted and quantified as previously described (Wang et al., 2014). For the extraction of IAA and trans-zeatin, about 1 g of plant material was frozen in liquid nitrogen and ground into powder. Next, 10 mL of isopropanol-HCl buffer solution (2:0.002, v/v) was added to the powder and shaken for 30 min at 4°C. Subsequently, dichloromethane (20 mL) was added, shaken for 30 min, and then centrifuged at 13 000 g for 5 min. After centrifugation, the lower organic phase was transferred to a 50-mL tube and evaporated in a constant stream of nitrogen. Each sample was kept in the dark and resolubilized in 400 µL methanol containing 0.1% formic acid, and then filtered using a 0.22 µm microfilter for HPLC-MS/MS analysis. For brassinolide measurement, 2 g of plant material was homogenized in liquid nitrogen with 10 mL 80% methanol and then incubated at 4°C for 2h in the dark. The homogenate was centrifuged at 10 000*g* for 5minat 4°C. The supernatant was loaded onto a Bond Elut C18 column (ODS; Agilent Technologies, Santa Clara, CA USA) that was eluted with 80% methanol. The eluate was further loaded onto strata-X column that was eluted with 80% methanol. The eluate solution was evaporated in a constant stream of nitrogen, re-dissolved in 200 mL methanol, and then filtered using a 0.22 µm microfilter for HPLC-MS/MS analysis. Three independent biological replicates, each with five technical replicates, were assayed for each hormone.

### Statistical Analysis

One-way analysis of variance (ANOVA) was used to evaluate the effect of simultaneous treatment of MC and GA_3_ on the expression of hormone-related genes using SPSS 17.0 software. Means were compared using the LSD at 0.05 level. Other data were subjected to independent Student's t-test at 0.05 (*) or 0.01 (**) probability levels.

## Results

### MC Shortened Cotton Seedling Internodes by Reducing Cell Division and Cell Elongation

**Figure 1:**
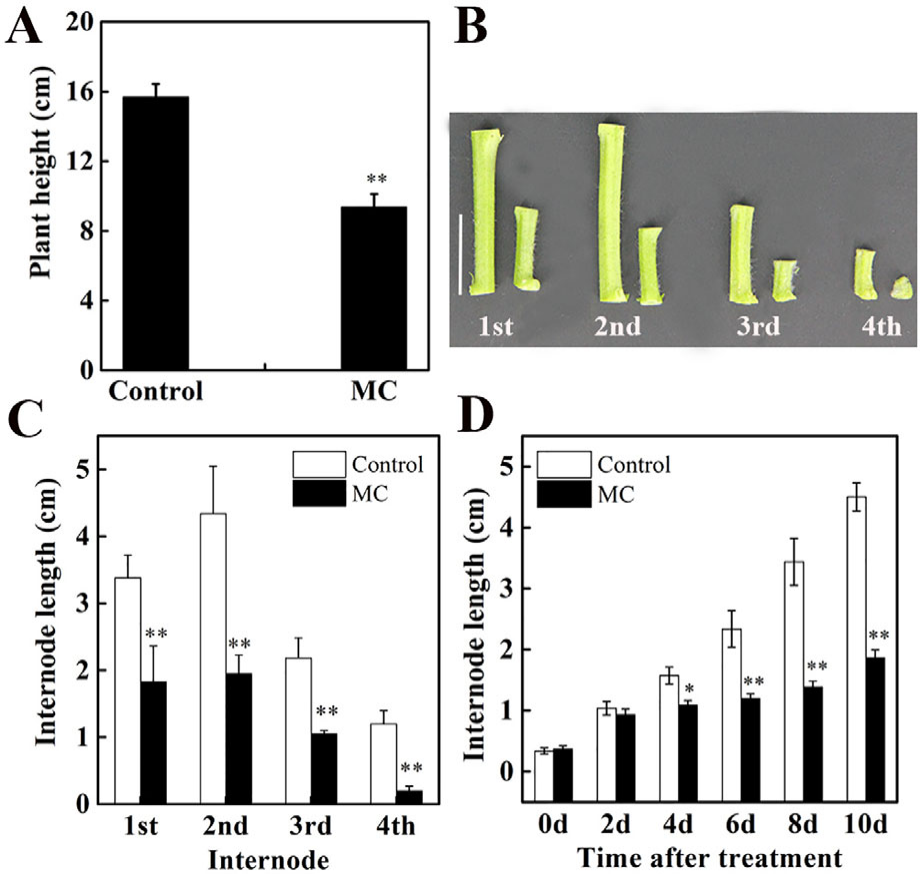
Effects of mepiquat chloride (MC) on plant height and internode length. Cotton seedlings at three-leaf-stage were treated with 80 mg/L MC by spraying. **(A)** Plant height, **(B)** internode phenotype and **(C)** internode length of control (deionized water) and MC-treated cotton seedlings at 10 days after MC treatment. **(D)** Dynamic changes in the second internode length of cotton seedlings after MC treatment. Error bars represent the SD of three biological and five technical replicates. Scale bars, 2 cm, *P < 0.05, **P < 0.01, Student's *t*-test.

Our previous study using cotton seedlings grown in the soil showed that the inhibition of internode elongation by MC was dose-dependent and 80 mg/L of MC was more efficient (Wang et al., 2014). In this study, cotton seedlings grown hydroponically were treated at three-leaf stage with foliar spraying of 80 mg/L MC. Ten days after MC treatment, plant height decreased by 40% compared to that of control plants (**Figure 1A**). The height of the first, second, third, and fourth internodes (from bottom to top of the stem) decreased by 46, 55, 52, and 83%, respectively (**Figures 1B, C**). The elongation of the second internode, the first visible and youngest sub-apical internode, was inhibited more severely than that of the first internode by MC application. The length of the second internode was reduced by 10, 31, 49, 53, and 55% at 2, 4, 6, 8, and 10 days after MC treatment, respectively (**Figure 1D**). The inhibition of internode elongation by MC was time-dependent. These results were consistent with our previous observations using cotton seedlings grown in the soil (Wang et al., 2014). Therefore, the second elongating internode at different time points after MC treatment was used for further study.

**Figure 2:**
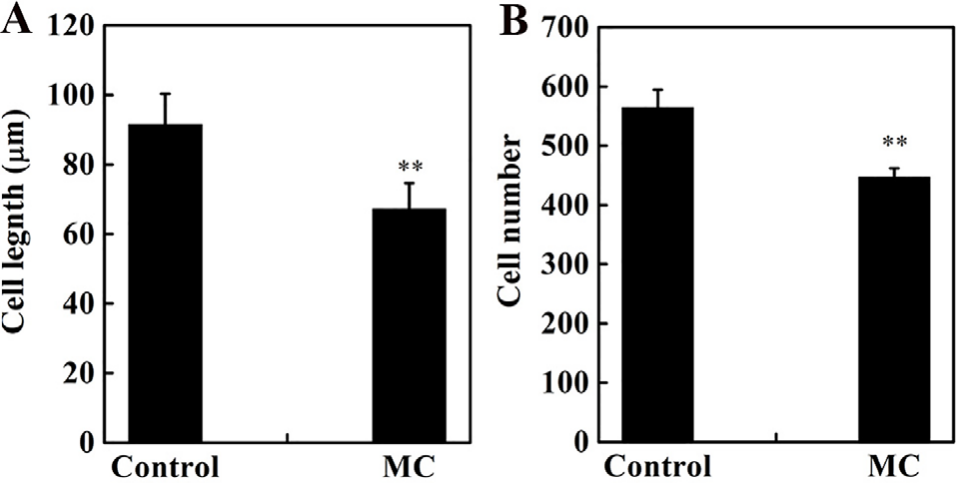
Effects of MC treatment on cell length **(A)** and cell number **(B)** from the second internode cortex of cotton seedlings at 10 days after treatment. Error bars represent the SD of three biological and five technical replicates. **P < 0.01, Student's *t*-test.

Internode elongation involves both cell division and cell elongation (Wang et al., 2018). Previously, we reported that cell elongation in the internode was inhibited by MC (Wang et al., 2014). To determine whether the cell division in the elongating internode was also inhibited by MC application, we evaluated cell number and cell length along the vertical axes of the second internode at 10 days after MC treatment. Consistent with our previous results (Wang et al., 2014), the longitudinal cells in the cortex and pith of MC-treated internodes were significantly shorter than those of control internodes (**Figure S1**), the average longitudinal cell length of MC-treated internodes was 27% shorter than that of the control internodes (**Figure 2A**). The cell number along the second internode of the MC-treated plants was 21% less than that of the control plants (**Figure 2B**). Therefore, MC shortened internodes by suppressing not only cell elongation but also cell division.

### Overview of the Transcriptome Profile of the Second Internode in Response to MC Treatment

To investigate the transcriptional regulatory mechanisms underlying the inhibition of internode elongation by MC, we analyzed the transcriptome profiles of the second internodes of cotton seedlings from MC-treated plants (0, 48, 72, and 96 h after MC treatment). In all, 12 RNA-Seq libraries were constructed and sequenced. An overview of the RNA-Seq sequencing reads derived from the 12 libraries is shown in **Table 1**. After adaptor sequence and low-quality reads were removed, each library generated 64.9 to 74.6 million clean reads (**Table 1**). All 12 libraries had a constant GC content of approximately 43% and high Q30 percentage of approximately 94%. The proportion of clean reads mapped to the cotton genome ranged from 94.8 to 96.2%, and that of the uniquely mapped reads ranged from 87.3 to 89.1%. For global comparison of the transcriptomes derived from 12 samples, principal component analysis among different time points and biological replicates was performed. PC1 clearly separated the control samples (0 h) from MC-treated samples (48, 72, and 96 h), and MC-treated samples at different time points showed less variance (**Figure S2**), indicating the expression of a substantial number of genes were altered to respond to MC treatment.

**Table 1:** Summary of sequenced reads and mapping results of RNA-Seq libraries.

Sample name^a^	Raw reads	Clean reads	Q30 (%)	GC content (%)	Total mapped	Multiple mapped	Uniquely mapped
0h -1	67855394	64904972	93.2	43.9	61507264 (94.8%)	4865201 (7.5%)	56642063 (87.3%)
0h -2	73708010	71773542	93.8	43.3	69032402 (96.2%)	5062153 (7.1%)	63970249 (89.1%)
0h -3	75369532	73516426	93.8	43.6	70106706 (95.4%)	5307252 (7.2%)	64799454 (88.1%)
48h -1	73297526	71221868	93.8	43.7	68271995 (95.9%)	5222539 (7.3%)	63049456 (88.5%)
48h -2	78997068	76151270	94.2	43.6	72804908 (95.6%)	5960774 (7.8%)	66844134 (87.8%)
48h -3	78116322	76378038	93.9	43.1	72961097 (95.5%)	5051227 (6.6%)	67909870 (88.9%)
72h -1	75917610	73986066	94.2	43.3	71032308 (96.0%)	5449090 (7.4%)	65583218 (88.6%)
72h -2	74496274	72546812	93.9	43.4	69372478 (95.6%)	5039603 (7.0%)	64332875 (88.7%)
72h -3	74790936	72704568	94.1	43.4	69631233 (95.8%)	5458006 (7.5%)	64173227 (88.3%)
96h -1	74650012	72340374	93.9	43.2	69382034 (95.9%)	5277424 (7.3%)	64104610 (88.6%)
96h -2	72959828	70537494	94.2	43.2	67509097 (95.7%)	5280269 (7.5%)	62228828 (88.2%)
96h -3	70121270	68244724	93.9	43.3	65098235 (95.4%)	4732830 (6.9%)	60365405 (88.5%)

a 0 h represents control; 48 h, 72 h, and 96 h represent 48 h, 72 h, and 96 h after mepiquat chloride treatment, respectively. Number 1, 2 and 3 at the end of the sample name represent three independent biological replicates.

### Differentially Expressed Genes (DEGs) in Response to MC Treatment

**Figure 3:**
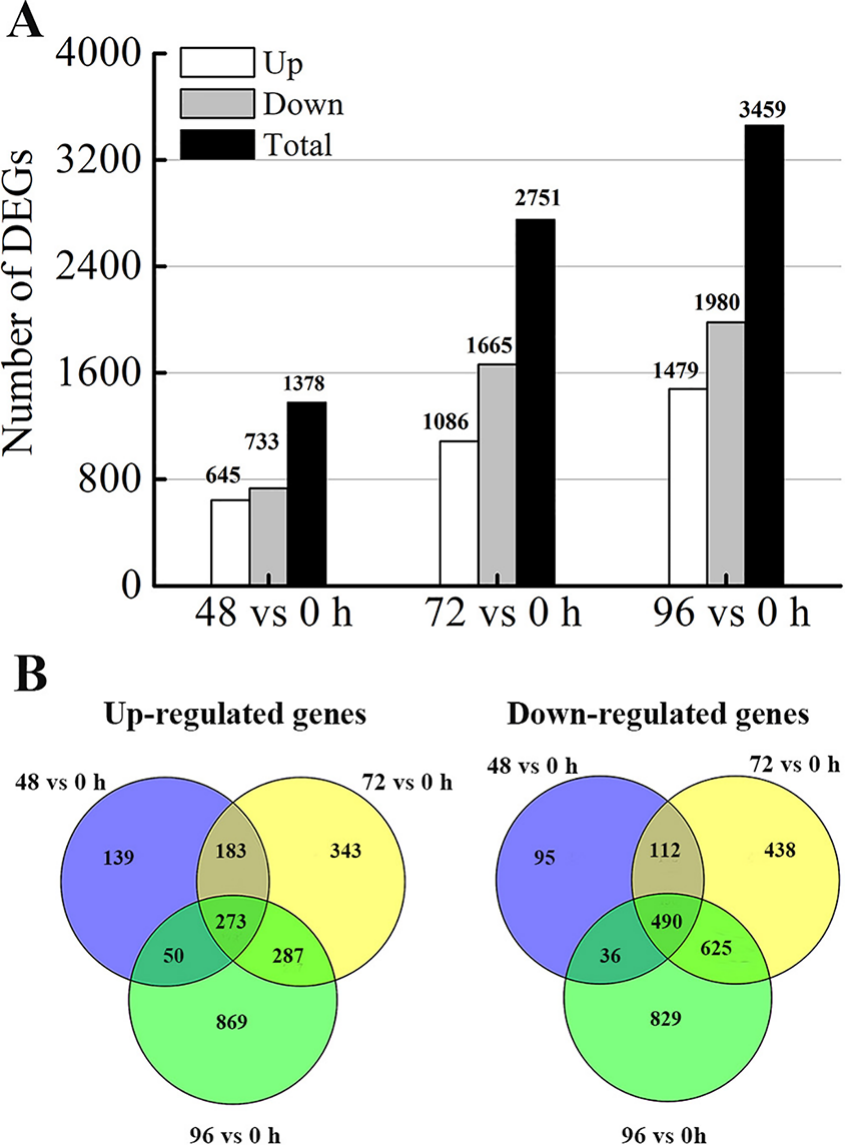
Differentially expression genes (DEGs) analysis. **(A)** Number of DEGs responding to MC-treatment (48 h, 72 h, 96 h vs. 0 h). **(B)** Venn diagram of DEGs at different time points after MC treatment.

To determine the gene expression changes in the internodes resulting from MC treatment, we normalized gene expression by using the fragments per kilobase of exon per million fragments mapped (FPKM) value. Transcripts with |log_2_ (fold change)| ≥ 1, false discovery rate (FDR) ≤ 0.05 and more than 1 FPKM in at least one sample of each comparison were considered as DEGs. Compared with that in the control (0 h), 1378 (645 upregulated and 733 downregulated), 2751 (1086 upregulated and 1665 downregulated), and 3459 (1479 upregulated and 1980 downregulated) DEGs were identified after MC treatment for 48, 72, and 96 h, respectively (**Figure 3A**). The numbers of both up- and down-regulated DEGs increased with time. As shown in the Venn diagrams (**Figure 3B**), 1058 (456 upregulated and 602 downregulated) DEGs were shared between 48 vs. 0 h and 72 vs. 0 h; 1675 (560 upregulated and 1115 downregulated) DEGs, between 72 vs. 0 h and 96 vs. 0 h; and 849 (323 upregulated and 526 downregulated) DEGs, between 96 vs. 0 h and 48 vs. 0 h. Among them, 273 and 490 genes were constantly induced or suppressed by MC treatment, respectively.

### Functional Analysis of the DEGs in Response to MC

To better understand the potential function of DEGs responding to MC application, we conducted Gene Ontology (GO) and Kyoto Encyclopedia of Genes and Genomes (KEGG) enrichment analyses for pairwise comparisons. GO enrichment was annotated according to biological process, cellular component, and molecular function (**Figure S3**). In general, for the biological process and molecular function groups, enrichment patterns of DEGs were similar at 48, 72, and 96 h. In the biological process category, the most dominant terms were “single-organism process,” “response to stimulus,” and “response to abiotic stimulus.” In the molecular function category, the most highly represented GO terms were associated with “binding,” “transcription factor activity, sequence-specific DNA binding,” and “nucleic acid binding transcription factor activity.” However, in the cellular component category, the top three enriched GO terms were different among the DEGs at the three time points: “chloroplast part,” “plastid part,” and “thylakoid” were dominantly enriched at 48 h; “cell part,” “cell,” and “cell periphery” were enriched at 72 h; whereas “chromatin,” “chromosomal part,” and “chromosome” were enriched at 96 h.

**Figure 4:**
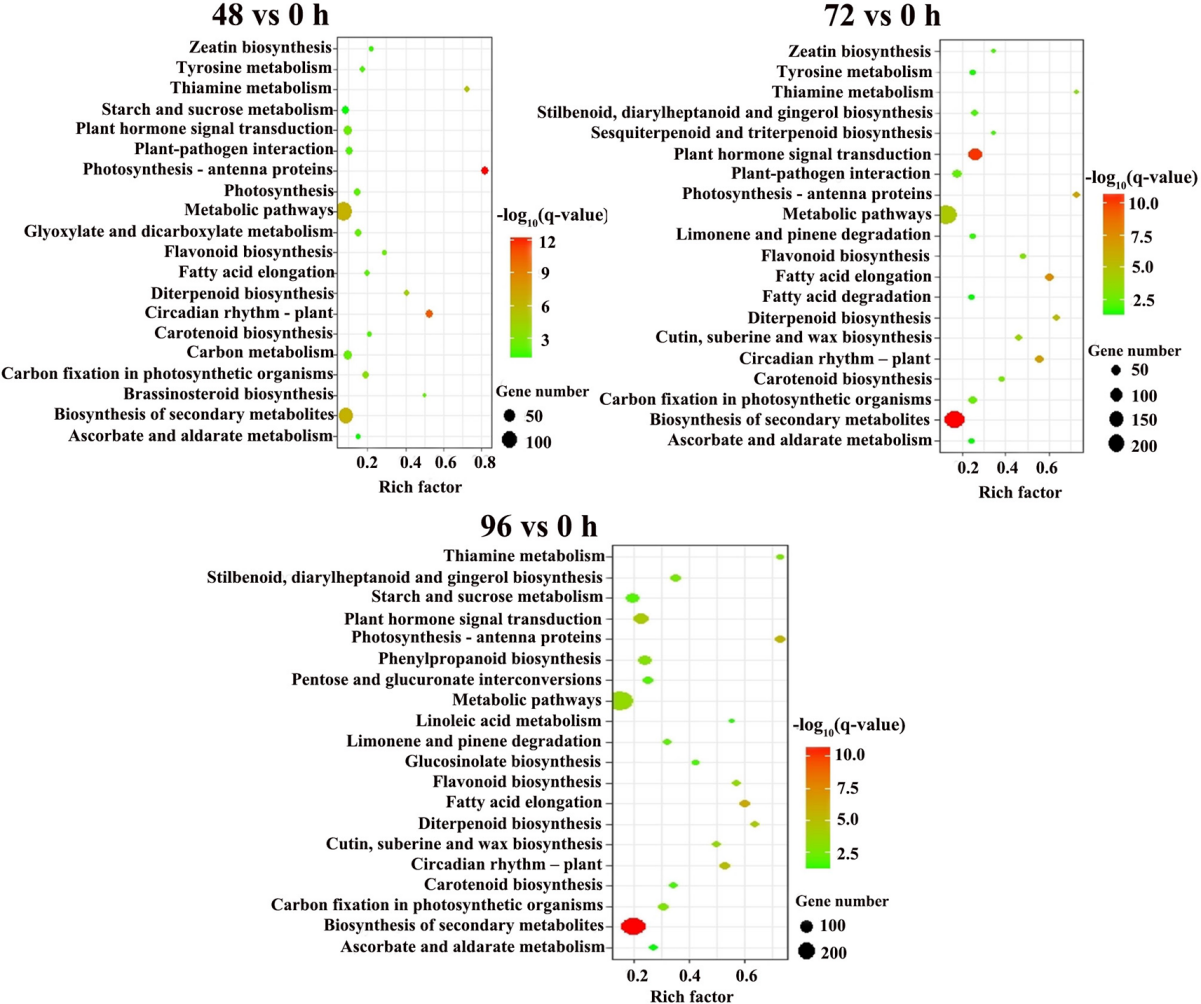
KEGG pathway enrichment analysis of DEGs between MC treatment and control. Y-axis shows the KEGG pathway. X-axis shows the Rich factor. The size and color of each point represents the number of genes enriched in a particular pathway and the -log_10_ (q-value), respectively, 0 h: control; 48, 72, and 96 h: 48 h, 72 h, and 96 h after MC-treatment.

For the KEGG pathway enrichment analysis, 89, 73, and 82 pathways were categorized from pairwise comparisons between 48 vs. 0 h, 72 vs. 0 h and 96 vs. 0 h, respectively (**Table S2**). The top 20 enriched KEGG pathways of each comparison are shown in **Figure 4**. The most significantly enriched KEGG pathways in the 48 vs. 0 h DEGs was photosynthesis-antenna proteins, followed by circadian rhythm-plant, biosynthesis of secondary metabolites, metabolic pathways, and thiamine metabolism. Five KEGG pathways were identified to be significant for the DEGs from the 72 vs. 0 h as well as 96 vs. 0 h comparison, which included biosynthesis of secondary metabolites, plant hormone signal transduction, fatty acid elongation, circadian rhythm-plant, and photosynthesis-antenna proteins.

### DEGs Related to Cell Cycle and Cell Expansion

Based on the reduced cell number and length in MC-treated internodes, we investigated the changes in the expression of genes related to cell cycle and cell wall. In total, 57 DEGs were found to be related to cell cycle and cell division (**Figure S4A** and **Table S3**). Interestingly, most of these genes were significantly downregulated, including cyclin genes (*CYCA1;1*, *CYCA2;2*, *CYCA2;3*, *CYCA3;2*, *CYCB2;4*, *CYCD3;1*, *CYCD3;2*, *CYCD4;1*, *CYCU1;1*, *CYCU2;1*, *CYCU4-1*), cyclin-dependent kinase B1;2 (*CDKB1;2*), cyclin-dependent kinase regulatory subunit 1 (*CKS1*), E2F transcription factor-like (*E2FF*), G2/mitotic-specific cyclin-1 (*CCNB1*), peptidyl-prolyl cis–trans isomerase (*FKBP65*), cell division control proteins (*CDC2*, *CDC7*, *CDC45*), cytochrome P450 78A (*CYP78A3*, *CYP78A5*, *CYP78A7*), syntaxin-related protein (*KNOLLE*), mitotic spindle checkpoint protein (*BUBR1*), MFP1 attachment factor 1 (*MAF1*), and meiotic nuclear division protein 1 (*MND1*). However, nine cell cycle-related genes were upregulated, such as cyclin-dependent kinase inhibitor (*KRP6* and *KRP7*) and cell number regulator 1 (*CNR1*; **Table S3**).

Cell wall architecture is a key determinant of plant growth. We identified 75 DEGs involved in the biosynthesis and modification of cell wall (**Figure S4B** and **Table S4**). Most of these genes were highly expressed in the control internodes, such as cell wall proteins [arabinogalactan peptides (*AGPs*) and fasciclin-like arabinogalactan proteins (*FLAs*)], α-expansins (*EXPAs*), and xyloglucan endotransglycolase/hydrolases (*XTHs*). Among these DEGs, 45 genes were significantly downregulated by MC, including three cellulose synthase-like protein G3 (*CSLG3*), one glucuronoxylan glucuronosyltransferase 7 (*IRX7*), two pectinesterase (*PME68*), five pectinesterase/pectinesterase inhibitor (*PMEI*), three galacturonosyltransferase-like (*GATL1*, *GATL7*, *GATL9*), four AGPs (*AGP20*, *AGP30*), four FLAs (*FLA2*, *FLA8*), three leucine-rich repeat extensin-like protein (*LRX2*, *LRX4*), one BURP domain-containing protein 17 (*BURP17*), one putative cell wall protein, six *EXPAs* (*EXPA4*, *EXPA8*, *EXPA15*), and twelve *XTHs* (*XTH2*, *XTH8*, *XTH9*, and *XTH32*). Notably, the expression of cell wall protein-encoding genes, *BURP17*, *LRX4*, *EXPAs* (*Gh_D10G1145*, *Gh_A10G2323*, and *Gh_D05G2934*), and *XTHs* (*Gh_D02G0220*, *Gh_A10G0146*, *Gh_A11G2885*, and *Gh_D11G3271*) showed more than 2-fold down-regulation at 96 h after MC treatment.

Cell division and expansion require the continuous uptake of water to maintain turgor pressure. Aquaporins (AQPs) play crucial roles in the transport of water and other small molecules across membranes in plants (Ding et al., 2019). According to sequence similarity and sub-cellular localization, AQPs are divided into five subgroups: plasma membrane intrinsic proteins (PIPs), tonoplast intrinsic proteins (TIPs), nodulin26-like intrinsic proteins (NIPs), small basic intrinsic proteins (SIPs), and X intrinsic proteins (XIP) (Li et al., 2014). PIPs can be further divided into PIP1 and PIP2, on the basis of sequence similarity. To assess whether and which subfamily members of AQPs are affected by MC treatment, we analyzed the differentially expressed AQPs. Our RNA-Seq data showed that some members of *PIPs* and *TIPs* were highly expressed in the internodes of control plants (**Figure S4C** and **Table S5**). In contrast, 19 *PIPs* and 7 *TIPs* were consistently suppressed after MC treatment, and only three *PIPs* were induced by MC (**Figure S4C**). The expression level of three *PIPs* (*Gh_A01G1843*, *Gh_D09G1409*, and *Gh_D01G2086*) and three *TIPs* (*Gh_D03G1253*, *Gh_D10G2205*, and *Gh_A10G1912*) was decreased by more than 2-fold. The decreased expression of *PIPs* and *TIPs* may have contributed to the inhibition of internode elongation by MC.

### DEGs Related to Hormone Metabolism and Signal Transduction

**Figure 5:**
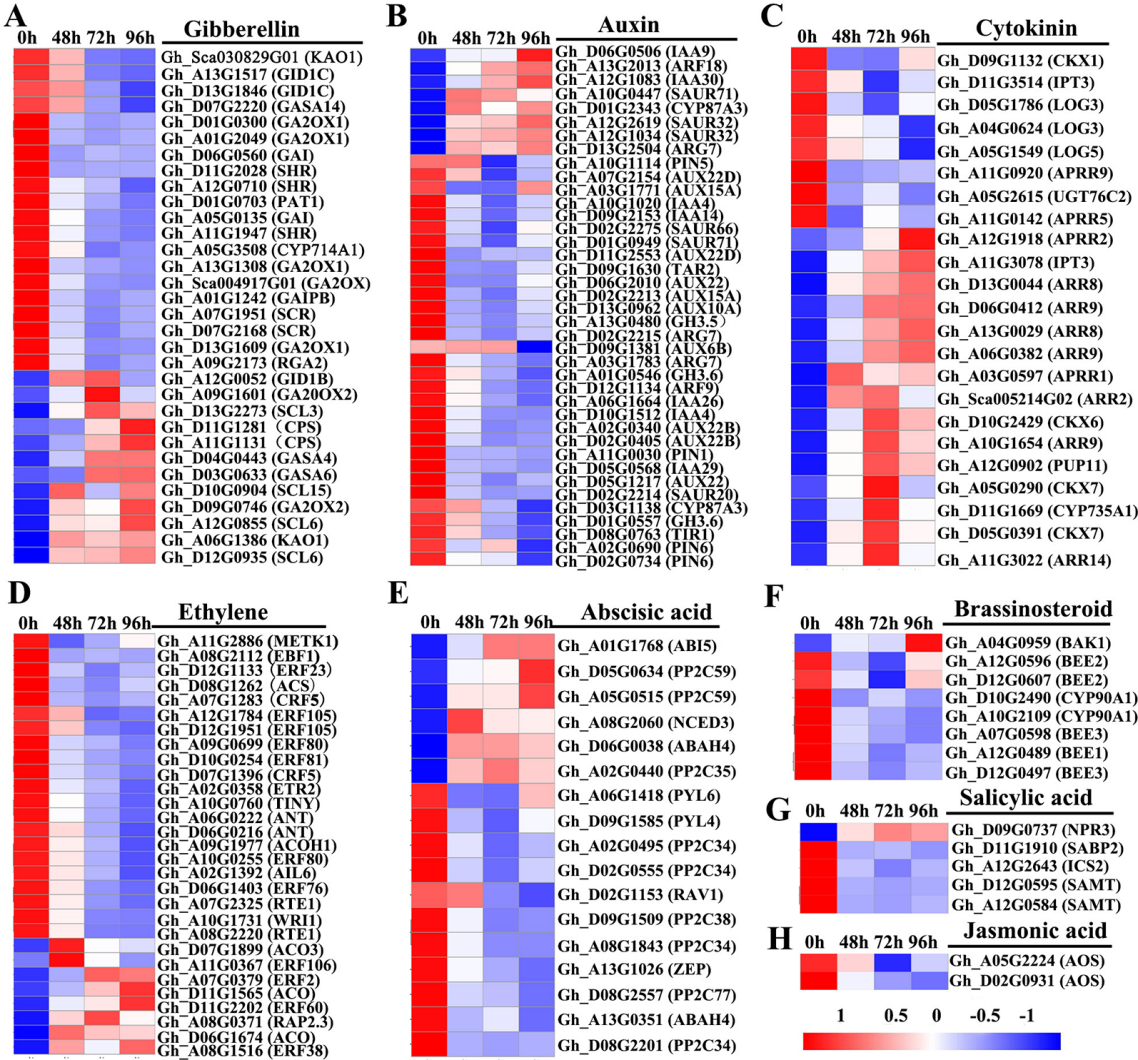
Heatmap of differentially expressed genes involved in plant hormone biosynthesis, transport, and signaling transduction in the second internode between MC treatment and control. **(A–H)** represent gibberellin , auxin, cytokinin, ethylene, abscisic acid, brassinosteroid, salicyclic acid and jasmonic acid, respectively. Gene expression level (FPKM) was normalized with Z-score. Blue indicates lower expression, and red indicates higher expression. 0 h: control; 48, 72, and 96 h 48 h, 72 h, and 96 h after MC-treatment.

To investigate the roles of hormones in the inhibition of internode elongation by MC, we identified DEGs involved in the phytohormone metabolism, transport, and signal transduction pathways by comparing the profiles between control samples and post MC treatment samples. About 155 hormone-related DEGs were detected after treatment with MC (**Figure 5** and **Table S6**). Most of these genes belonged to auxin metabolism and response pathways, followed by GA, ethylene, CK, and ABA; relatively fewer DEGs were related to salicylic acid (SA) and jasmonic acid (JA) (**Figure 5**). Most of the DEGs showed decreased expression except for the CK pathway-associated DEGs, which were generally induced by MC treatment (**Figure 5**).

Thirty-two DEGs related to GA were found to be responsive to MC application (**Figure 5A**). In the GA metabolism pathway, seven DEGs were found to be downregulated after MC treatment, including one biosynthetic gene (*KAO*, *Gh_Sca030829G01*) and six catabolic genes (five *GA2ox1* and one *CYP714A1*), whereas genes encoding another KAO (*Gh_A06G1386*), two ent-copalyl diphosphate synthase (*KS*), one GA 20 oxidase 2 (*GA20ox2*), and one GA 2 oxidase2 (*GA2ox2*) were upregulated by MC. In the GA signal transduction pathway, 14 genes encoding GRAS family regulatory proteins, including DELLA (*GAI*, *RGA2*, *SCR*, *SHR*, and *PAT1*), and genes encoding GID1 were downregulated at different time points (**Table S6**).

Of the 39 DEGs related to auxin pathway, the genes related to auxin biosynthesis (*TAR2*), catabolism (*GH3*), signaling (*TIR1*, *ARF9*, *AUX22*, *AUX6B*, *AUX15A*, *AUX10A*, *IAA4*, *IAA14*, *IAA29*, and *IAA26*), and transport (*PIN1*, *PIN5*, and *PIN6*) were downregulated at different time points (**Figure 5B**). Eight genes encoding auxin-responsive proteins, including *SAUR66*, *SAUR20*, and *SAUR71*, were also significantly downregulated (**Table S6**).

Expression of 23 DEGs involved in CK biosynthesis, modification, transport, and signaling was altered in response to MC (**Figure 5C**). *IPT3* (*Gh_A11G3078*), encoding a rate-limiting enzyme in CK biosynthesis, was upregulated by 2.9-, 3.2-, and 3.6-fold at 48, 72, and 96 h after MC treatment, respectively, whereas another *IPT3* (*Gh_D11G3514*) was downregulated only at 48 h after treatment. *Gh_D11G1669*, a cytokinin hydroxylase (*CYP735A1*), was significantly induced by MC treatment. The expression of four orthologs of CYTOKININ OXIDASES/DEHYDRO-GENASES (*CKX6* and *7*) involved in the irreversible degradation of CKs was significantly upregulated at different time points, and only *CKX1* was downregulated. The gene encoding CK transporter purine permease 11 (*PUP11*) was also upregulated. The expression of *UGT76C2*, which encodes a glycosyltransferase that catalyzes the *N*-glycosylation of CK, was downregulated. The expression of three genes (*LOG3* and *LOG5*) encoding CK-activating enzymes was significantly decreased, especially at 96 h after MC treatment. ARRs are associated with CK signal transduction pathway; nine induced ARR genes (two B-type *ARRs*, five A-type *ARRs*, and two *B-ARR-like* genes) and four suppressed *ARR-like* genes were identified after MC treatment (**Table S6**).

Six DEGs were found to be related to ethylene biosynthesis (**Figure 5D**). Among them, a gene encoding one *S*-adenosyl methionine (SAM) synthase, one 1-aminocyclopropane-1-carboxylate (ACC) synthase (*ACS*), and one homolog of ACC oxidase (*ACO*) were significantly downregulated in response to MC; interestingly, the *ACO* homolog was downregulated by 2.0- and 5.2-fold at 72 and 96 h, respectively. However, three ACO genes were upregulated, especially at 48 h after MC treatment. In the ethylene-response pathway, eighteen DEGs were found to be downregulated by MC, including one *ETR2*, one *EBF1*, fourteen ethylene-responsive TFs (*ERF*, *WRI1*, *TINY*, *AIL6*, *ANT*, *RAP23*, and *CRF5*), and two REVERSION-TO-ETHYLENE SENSITIVITY1 (*RTE1*) genes (**Table S6**).

In the ABA metabolism pathway, one gene encoding 9-cis epoxy carotenoid dioxygenase3 (NCED3) and one ABA 8ʹ-hydroxylase (ABAH) encoding gene were upregulated by MC, whereas one zeaxanthin epoxidase (*ZEP*) gene and two *ABAHs* were consistently suppressed (**Figure 5E**). Fifteen ABA signaling-related genes were identified as DEGs, with eight downregulated genes (*PYL4*, *PYL6*, *PP2C27*, *PP2C34*, and *PP2C38*) and five upregulated ones (*ABI*5, *PP2C35*, and *PP2C59*) (**Table S6**).

Two *CYP90A1*/*CPD* (constitutive photomorphogenesis and dwarfism) genes involved in BR biosynthesis were significantly downregulated by MC treatment (**Figure 5F**). However, *Gh_A04G0959* (*BAK1*), encoding a receptor kinase mediating BR signaling, was upregulated 96 h after MC treatment. Five BR signaling components BR-ENHANCED EXPRESSION (*BEE*) were significantly downregulated by MC treatment, including one *BEE1*, two *BEE2*, and two *BEE3* (**Table S6**).

The expression of *Gh_A12G2643*, isochorismate synthase 1 (ICS1) involved in SA biosynthesis; *Gh_D12G0595* and *Gh_A12G0584*, salicylate carboxymethyltransferase (SAMT) related to the conversion of SA to SA methyl ester; and *Gh_D11G1910*, salicylic acid-binding protein 2 (SABP2) converting MeSA to SA, was suppressed by MC (**Figure 5G**). In the SA response pathway, one *NPR3*, a SA receptor, was significantly upregulated. In the JA biosynthesis and signaling pathway, only two allene oxide synthase *AOS*/*CYP74A* genes were downregulated by MC treatment (**Figure 5H**).

### DEGs Related to Lignin and Flavonoid Metabolism

KEGG enrichment showed that DEGs involved in secondary metabolism were overrepresented in response to MC treatment. Therefore, we analyzed the DEGs related to lignin and flavonoid metabolism, which are associated with plant growth (Dare et al., 2013; Muro-Villanueva et al., 2019). The biosynthetic pathways leading to lignin and flavonoid diverge at the common intermediate *p*-coumaroyl CoA. A total of 37 genes involved in lignin biosynthesis were differentially expressed in the internodes between MC-treated and control plants (**Figure S5A** and **Table S7**). Among these genes, 22 DEGs were downregulated by MC, including two 4-coumarate-CoA ligase (*4CL*), one 4-coumarate-CoA ligase-like (*4CLL*), two caffeoylshikimate esterase (*CSE*), four shikimate *O*-hydroxycinnamoyltransferase (*HCT*), one caffeic acid 3-*O*-methyltransferase (*COMT*), one cinnamyl alcohol dehydrogenase (*CAD*), one cinnamoyl-CoA reductase (*CCR*), four laccase (*LAC*), and six peroxidase (*PRX*). Notably, one *HCT* showed more than 2.5-fold down-regulation after MC treatment. Fifteen DEGs were upregulated, including one *4CL*, two *CSE*, two *COMT*, one *CAD*, one *LAC*, and eight *PRX*.

In total, 14 DEGs involved in flavonoid biosynthesis were identified (**Figure S5B** and **Table S7**). Among these DEGs, 10 were downregulated by MC, including two *CHS*, one leucoanthocyanidin dioxygenase (*LDOX*), two flavonol synthase (*FLS*), one flavanone 3-dioxygenase, one flavonoid 3-*O*-glucosyltransferase, two anthocyanin 5-aromatic acyltransferase, and one flavonol sulfotransferase-like. The catalysis of CHS is the initial step of the flavonoid biosynthesis pathway. Interestingly, two *CHS* genes showed significant down-regulation at different time points after MC treatment, with more than 3-fold down-regulation both at 72 and 96 h.

### Differentially Expressed TFs

The changes in gene expression level are known to be closely related to the alterations of TFs. We identified 497 differentially expressed TFs that belong to 46 different families, including MYB, Orphans, bHLH, AP2-EREBP, HB, WRKY, NAC, bZIP, GRAS, AUX/IAA, GRF, and TCP, in response to MC treatment (**Table S8**). Some important TFs are shown in **Figure S6**. Most of the MYBs were downregulated, including genes functioning in plant circadian clock and ABA accumulation (Adams et al., 2018) and those associated with the ABA signal pathway (Ni et al., 2018). The bHLH TFs play important roles in the control of cell elongation. Five *BEEs* (*BEE1*, *BEE2*, and *BEE3*), BR early-response genes and associated with BR-regulated cell elongation (Friedrichsen et al., 2002), and four *PREs* (*PRE5*, *PRE6*) were significantly downregulated. The expression of AP2/EREBP family TFs was altered in MC-treated internodes. Twelve ERF genes involved in ethylene signaling and response pathway were also downregulated in response to MC. Two *ANT* and *CRF5* genes each were downregulated. Among Orphans family TFs, fourteen *BBX* (*BBX19*, *BBX24*, *BBX32*) TFs were significantly downregulated by MC. One *ETR2*, an ethylene receptor, was significantly downregulated. In contrast, ten *CONSTANS*, positive regulators of photoperiodic flowering, and seven *ARR* (*ARR2*, *ARR8*, *ARR9*, and *ARR14*) genes were significantly upregulated. Moreover, 14 NAC TFs were upregulated in response to MC (**Table S8**). The HB family TFs such as *ATHB*, *KNAT6*, and *HAT* were significantly downregulated, except for *HAT22*, which was upregulated. The majority of WRKY family TFs was upregulated (**Table S8**). Nine bZIP members, were significantly downregulated (**Table S8**). The transcription level of all GRF family TFs decreased gradually after MC treatment. Remarkably, most of *GRF* genes showed more than 2-fold down-regulation 96 h after MC treatment compared with that in the control. Six TCP TFs were downregulated.

### Validation of RNA-Seq Data Using qRT-PCR

**Figure 6:**
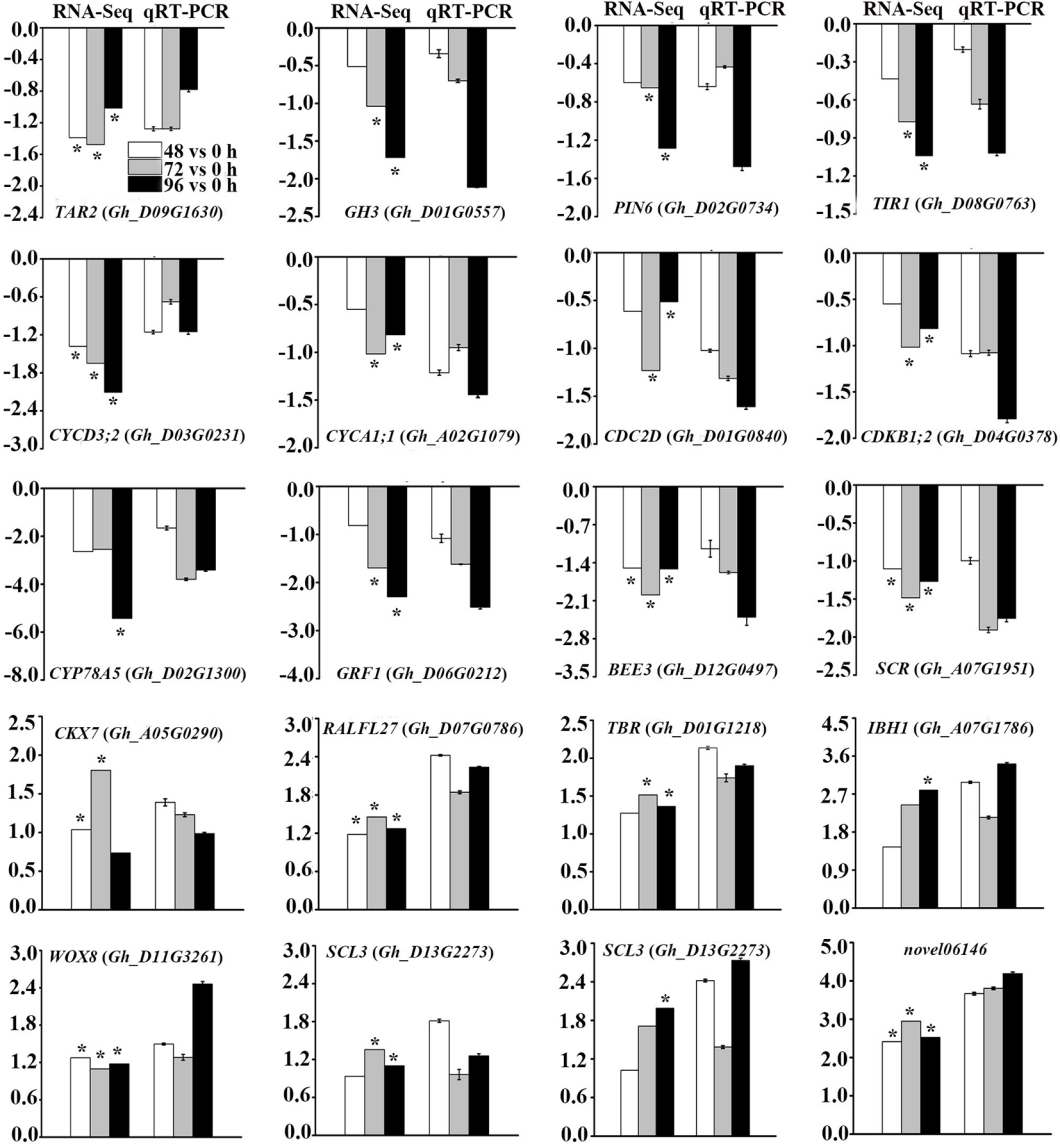
Validation of DEGs from RNA-Seq analysis by quantitative reverse transcription polymerase chain reaction (qRT-PCR). Y-axis represents the log_2_ fold change values at 48 h, 72 h, and 96 h after MC treatment compared to control (0 h) (RNA-Seq), or -delt delt Ct (qRT-PCR). * Indicates significantly regulated by RNA-Seq analysis. Error bars represent the SD of three biological and two technical replicates.

To validate the reliability of RNA-Seq data, we randomly selected 19 DEGs related to phytohormone biosynthesis and signal transduction pathways, cell cycle-related genes and TFs for qRT-PCR analysis (**Figure 6**). In addition, a novel gene which showed more than two-fold up-regulation at different time points after MC treatment was selected to be validated. The expression profiles of these genes from both qRT-PCR and RNA-Seq analyses were highly consistent, indicating the reliability of the RNA-Seq data.

### Changes in Endogenous Hormone Content in the Second Internode After MC Treatment

**Figure 7:**
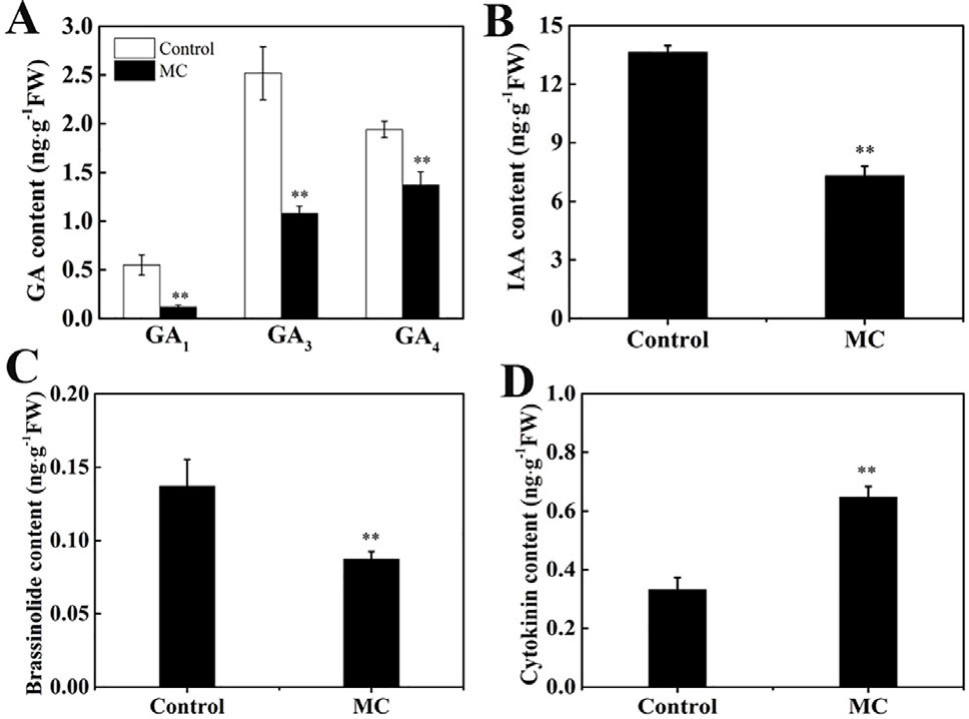
Effects of MC treatment on the contents of endogenous phytohormone in the second internode of cotton seedlings 6 days after treatment. **(A)** GA_1_, GA_3_, and GA_4_, **(B)** IAA, **(C)** brassinolide and **(D)** cytokinin. Error bars represent the SD of three biological and five technical replicates. **P < 0.01, Student's *t*-test.

Previously, we showed that MC significantly reduced endogenous GA_3_ and GA_4_ levels during internode elongation (Wang et al., 2014). Numerous genes associated with hormone biosynthesis and signal transduction pathway were altered by MC treatment. To investigate whether MC affects the contents of other endogenous hormones, we quantified IAA, BR, and CK (*trans*-zeatin) as well as different types of GA (GA_1_, GA_3_, and GA_4_) levels in the second internode of control and MC-treated plants. The contents of GA_1_, GA_3_, and GA_4_ were reduced by 78, 57, and 29% in MC-treated plants 6 days after treatment, respectively (**Figure 7A**), confirming our previous results. Similarly, endogenous IAA and brassinolide (BL), the most biologically active BRs, were reduced by 46% and 36%, respectively (**Figures 7B, C**). In contrast, endogenous CK level was increased by 95% in MC-treated plants (**Figure 7D**). These data indicate that the dwarfism caused by MC is closely related to the reduction of endogenous GA, IAA, and BR content.

### Exogenous GA Can Antagonize the Effect of MC on Transcription of Hormone-Related Genes

**Figure 8:**
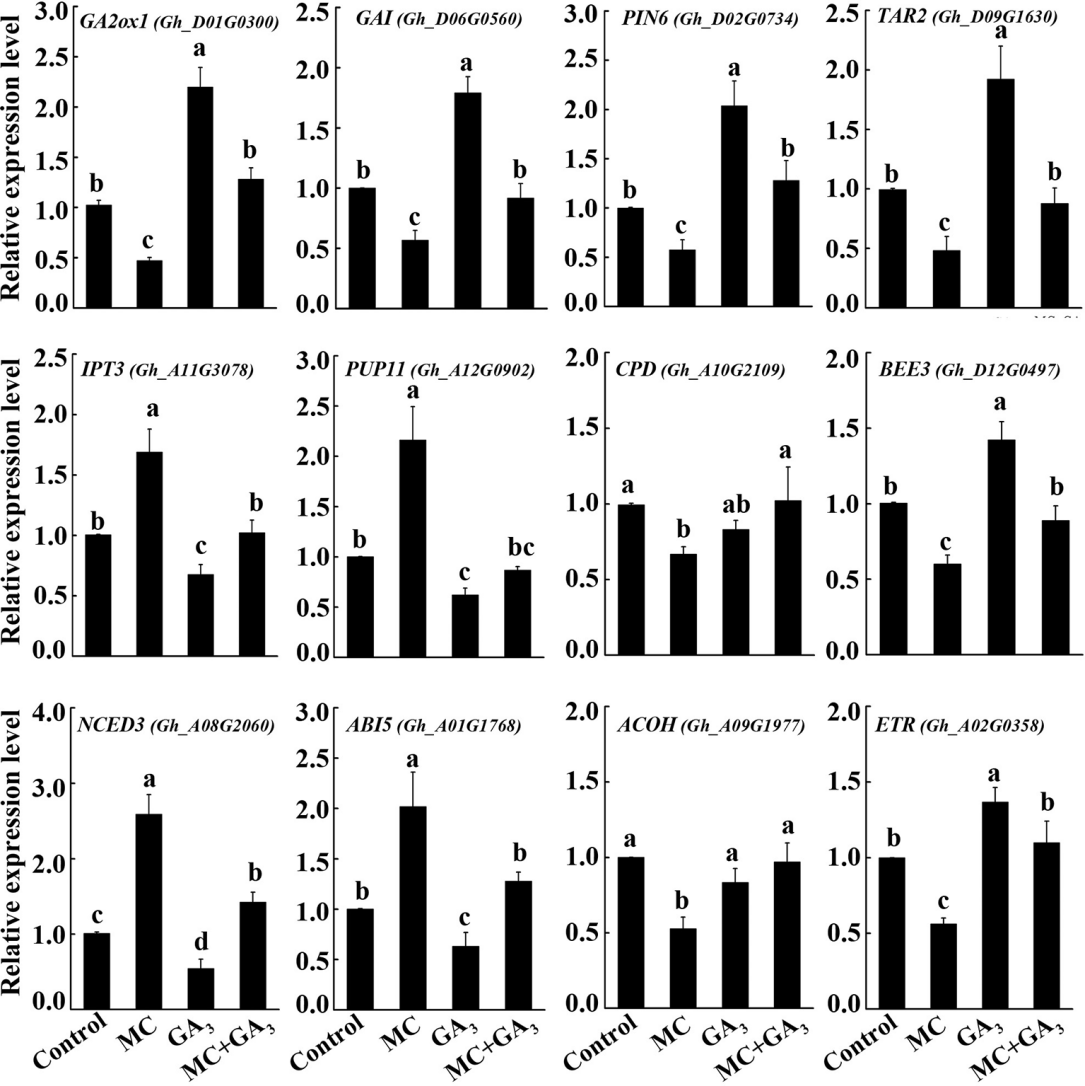
Exogenous GA antagonize the effect of MC on the expression of hormone-related genes. Cotton seedlings at three-leaf-stage were treated with deionized water (control), 80 mg/L MC, 100 μM GA_3_, and combination of 80 mg/L MC and 100 μM GA_3_ (MC+GA_3_) by spraying. Samples were collected at 72 h after treatments. Expression of twelve genes of GA (*GA2ox1* and *GAI*), auxin (*PIN6* and *TAR2*), cytokinin (*IPT3* and *PUP11*), brassinosteroid (*CPD* and *BEE3*), abscisic acid (*NCED3* and *ABI5*), and ethylene (*ACOH* and *ETR*) metabolism, transport or signaling pathways were tested by qRT-PCR. *GhActin4* was used as an internal control to normalize the gene expression. Gene expression in the control samples was set as 1.0, fold-changes of RNA transcripts were calculated by the 2^−△△Ct^. Error bars represent the SD of three biological and two technical replicates. Small letters above the bars indicate statistically significant differences by One way ANOVA (LSD, *P* < 0.05).

The crosstalk between GA and other hormones has been reported (Wang et al., 2015b; Liu et al., 2018; Zhang et al., 2019). To verify whether the altered transcription of many hormone-related genes by MC is GA dependent or not, three-leaf-stage seedlings were treated with MC, GA_3_ or both MC+GA_3_ by foliar spraying. The expression changes of twelve genes from GA (*GA2ox1* and *GAI*), auxin (*PIN6* and *TAR2*), CK (*IPT3* and *PUP11*), BR (CPD and *BEE3*), ABA (*NCED3* and *ABI5*) and ethylene (*ACOH* and *ETR*) metabolism, transport or signaling pathways were determined using qRT-PCR. As shown in **Figure 8**, most of these genes responded oppositely to MC treatment compared to exogenous GA treatment. MC significantly reduced the expression levels of *GA2ox1*, *GAI*, *PIN6*, *TAR2*, *CPD*, *BEE3*, *ACOH*, and *ETR*, while induced the expression of *IPT3*, *PUP11*, *NCED3*, and *ABI5*; conversely, exogenous GA_3_ application increased the expression of *GA2ox1*, *GAI*, *PIN6*, *TAR2*, *BEE3*, and *ETR*, and decreased that of *IPT3*, *PUP11*, *NCED3*, and *ABI5*, *CPD*, and *ACOH* did not respond to exogenous GA. Interestingly, most of these genes under simultaneous MC+GA_3_ treatment showed similar expression levels to those in control samples. These results clearly demonstrate that MC does not directly affect other plant hormones, but through inhibition of the GA biosynthesis.

## Discussion

MC has been the most successful and widely used plant growth regulator in cotton; it allows to compact plants by reducing internode length and leaf size (Reddy et al., 1990; Siebert and Stewart, 2006; Ren et al., 2013). Previously, we showed that MC reduced the synthesis of GA, resulting in the suppression of cell elongation in the internodes of cotton seedlings (Wang et al., 2014). In this study, we analyzed the global transcriptional changes in the second elongating internodes of cotton seedlings in response to MC to elucidate the other potential mechanisms underlying MC-induced internode elongation.

### MC Reduced Internode Elongation by Inhibiting Cell Division and Expansion

As previously reported (Reddy et al., 1990; Siebert and Stewart, 2006; Ren et al., 2013; Wang et al., 2014), MC significantly reduced plant height by shortening internode length (**Figure 1**). MC has been shown to limit internode elongation mainly by obstructing cell elongation (York, 1983; Wang et al., 2014). Our histological observation indicated that cell number and length of internodes were both significantly reduced by MC (**Figures 2** and **S1**). Consistent with the results of microscopic analysis, transcriptome data showed that numerous genes related to cell cycle and cell wall were altered by MC treatment. In plants, the cell cycle is mainly regulated by CDKs and cyclins (Sablowski and Carnier-Dornelas, 2013). Our study showed that B-type CDKs; CKS1; A-, B-, D-, and U-cyclins; and G2/mitotic-specific cyclins were downregulated by MC (**Figure S4A**). The upregulation of cell cycle inhibitor genes (*KRP6* and *KRP7*) by MC could also contribute to the reduced cell division (**Figure S4A**). Moreover, five genes encoding cell division control proteins were downregulated, these proteins primarily target cell cycle regulators for proteolysis, (**Figure S4A**; Baek et al., 2011). The *CYP78A* family members have been shown to control reproductive organ growth and seed development by promoting cell proliferation in Arabidopsis (Adamski et al., 2009; Fang et al., 2012) and wheat (Ma et al., 2015a; Ma et al., 2015b), In this study, six *CYP78A* family members showed more than 2-fold downregulation 96 h after MC treatment (**Figure S4A**), suggesting that they might control internode elongation by regulating cell proliferation in cotton. A cotton *CNR1* was significantly upregulated in MC-shortened internodes (**Figure S4A**), consistent with the previous study that overexpression of *CNR1* reduced cell number in the internode of maize (Guo et al., 2010).

Cell elongation requires the selective loosening of cell walls (Wolf, 2017). EXPAs and XTHs are two types of important cell wall proteins that are involved in cell elongation. EXPAs can break the hydrogen bonds linking cellulose and hemicellulose, especially xyloglucan, thereby loosening the cell wall (Marowa et al., 2016). XTHs can cleave and reattach xyloglucan polymers (Atkinson et al., 2009). In our previous study, we showed that MC suppressed the expression levels of *GhEXP* and *GhXTH2* in cotton internodes within 2–10 d after treatment (Wang et al., 2014). In this study, six *EXPs* and twelve *XTHs* were found to be significantly downregulated by MC (**Figure S4B**). In addition, other cell wall-related genes such as *PME*s, *FLA*s, and *AGP*s were also downregulated by MC treatment (**Figure S4B**), contributing to the inhibition of internode elongation. PMEs catalyze the demethyl esterification of pectin and regulate pectin reconstruction, thereby increasing the extension of cells (Wolf, 2017). High PME activity was required for cell elongation in germinating shoots (Jeong et al., 2015). AGPs have been reported to be involved in cell elongation (Coll-Garcia et al., 2004; Yang et al., 2014). FLAs, cell surface glycoproteins, belonging to AGPs, are important for normal cell expansion (Shi et al., 2003).

Cell growth is tightly related to the regulation of hydraulic and turgor pressure, which is associated with AQPs (Ding et al., 2019). The significance of PIPs and TIPs in tissue elongation has been shown by a positive correlation between gene expression and cell expansion in leaves, hypocotyls, roots, reproductive organs, and fruits (Chaumont and Tyerman, 2014). In this study, most *PIPs* and *TIPs* exhibited reduced expression in the internodes shortened by MC treatment (**Figure S4C**), which further affected internode elongation.

### MC Remarkably Altered the Biosynthesis and Signaling Transduction of Multiple Hormones in a GA-Dependent Manner

Previously, we showed that MC suppressed the expression of GA biosynthesis genes (*GhCPS*, *GhKS*, *GhGA20ox*, and *GhGA3ox*), GA catabolism genes (*GhGA2ox*), and signal transduction genes (*GAI*) (Wang et al., 2014). In this study, RNA-Seq profiles showed similar expression changes in GA metabolism and signaling genes. The majority of GA associated DEGs were downregulated by MC, including the GA biosynthesis gene (*KAO1*), GA catabolism genes (*GA2ox1*, *CYP714A1*), and GA signal transduction genes (*RGA2*, *GAI*, *GID1*) (**Figures 5A** and **8**). Endogenous GA_1_, GA_3_, and GA_4_ contents were remarkably reduced by MC 6 days after treatment (**Figure 7A**), consistent with the expression change of GA associated genes and our previous study (Wang et al., 2014). The bioactive GA homeostasis are maintained through a number of feedback and feed forward mechanisms (Wang et al., 2015b; Zhang et al., 2019). In this study, exogenous GA_3_ induced the expression of *GA2ox1* and *GAI* (**Figure 8**), in accordance with previous reports that *GA2ox* and *DELLAs* are up-regulated by exogenous GA (Wang et al., 2015b; Zhang et al., 2019).

Auxin also plays important roles in the determination of plant height by regulating cell expansion and cell division. The levels of free active auxin are determined by biosynthesis, conjunction, and polar transport in plant organs. Our RNA-Seq data showed that the expression of genes related to auxin biosynthesis (*TAR2*) and conjunction (*GH3*) were remarkably decreased by MC (**Figures 5B** and **8**). The decreased expression of *PIN* family genes (*PIN1*, *PIN5*, and *PIN6*) suggested that auxin transport and homeostasis were disrupted in cotton internodes by MC treatment (**Figures 5B** and **8**). Consistent with the decreased biosynthesis and transport of auxin, reduced IAA levels were observed in MC-treated internodes 6 days after treatment (**Figures 5B** and **7B**). Furthermore, the down-regulation of auxin signaling genes such as auxin receptor *TIR1*, auxin response factor *ARF*, and *AUX/IAA* transcriptional repressors indicated that auxin signal transduction was somehow blocked in cotton internodes by MC. Similarly, the inhibited expression of auxin-related genes were also observed in *Agapanthus praecox* (Zhang et al., 2015) and *Jatropha curcas* (Seesangboon et al., 2018), and in litchi by uniconazole (Ohnishi et al., 2006). Opposite to the downregulation by MC, we found that exogenous GA_3_ upregulated the expression of *TAR2* and *PIN6* (**Figure 8**), similar to previous studies in *Populus* (Björklund et al., 2007), *Arabidopsis* (Li et al., 2014), and *Eucalyptus grandis* (Liu et al., 2018). Under simultaneous MC+GA_3_ treatment, the expression of *TAR2* and *PIN6* showed no obvious difference compared with controls, suggesting that exogenous GA can offset the reduced GA content by MC. These results indicate that the repression of auxin-related genes by MC is through the inhibition of GA biosynthesis.

BRs are essential for stem elongation, and BR-deficient mutants exhibit characteristic dwarfism and compact stature (Velde et al., 2017). CPD is crucial for BR biosynthesis (Ohnishi et al., 2006). In this study, MC significantly inhibited the expression of two *CPDs*, as well as caused a remarkable reduction in BL content in MC-treated internodes (**Figures 5F**, **7C**, and **8**). *BEEs* are associated with BR signal transduction, the *bee1 bee2 bee3* triple mutant Arabidopsis showed similar phenotype to BR mutants (Friedrichsen et al., 2002). Overexpression of *BEE3-like* gene in poplar increased plant height and internode length (Noh et al., 2015). As expected, MC significantly downregulated *BEE1*, *BEE2*, and *BEE3*, whereas upregulated *BAK1*, this is probably due to the fact that BAK1 is shared with other signaling pathways such as plant immunity (Postel et al., 2010). Complicated crosstalk exists between BR and GA. Studies showed that the crosstalk between BR and GA differed depending on hormone concentrations, development stages, tissues, and plant species (Tong and Chu, 2016; Unterholzner et al., 2016). We found that exogenous GA_3_ increased the expression of *BEE3* but did not affect that of *CPD* (**Figure 8**). Similar to auxin-related genes, exogenous GA_3_ abolished the repression of MC on the expression of *CPD* and *BEE3*, indicating MC affects BR synthesis in a GA-dependent manner.

GAs and CKs are known to antagonistically regulate multiple developmental processes such as hypocotyl and internode elongation (Weiss and Ori, 2007). Unlike GA, auxin, and BR, most CK-related DEGs were upregulated by MC (**Figure 5C**). MC-induced increase of CK (trans-zeatin) content was consistent with the up-regulation of CK biosynthesis (*IPT3* and *CYP735A1*) and transport genes (*PUP11*) in cotton internodes (**Figures 5C**, **7D**, and **8**). This correlated well with other studies that other GA biosynthesis inhibitors such as PAC, uniconazole, and Pro-Ca increase CK levels in different plant tissues (Grossmann et al., 1994; Upreti et al., 2013; Liu et al., 2015). Active CKs are degraded mainly by CKX, which can be induced by exogenous CK (6-BA) owing to feedback mechanisms (Kieber and Schaller, 2018; Ni et al., 2018). Therefore, the MC mediated upregulation of *CKX1*, *CKX6*, and *CKX7* might be attributed to the increased CK levels in cotton internodes. A-type ARR genes are the primary responsive genes in CK signaling pathway and can be transcriptionally induced in response to cytokinin (Kieber and Schaller, 2018). Nine *ARR* genes were upregulated by MC in cotton seedlings (**Figure 5C**), suggesting that MC affects not only CK levels but also its signaling in cotton internodes. Application of exogenous GA_3_ led to repressed expression of *IPT3* and *PUP11* (**Figure 8**), this agree well with the previous studies that GA down-regulated the expression of CK-signaling-related genes in apple (Zhang et al., 2019) and *Camellia sinensis* (Di et al., 2019), and inhibited CK responses in Arabidopsis (Greenboim-Wainberg et al., 2005). The MC-induced expression of *IPT3* and *PUP11* was significantly inhibited by combined MC and GA_3_, suggesting MC affected CK biosynthesis, transport, and signaling in a GA-dependent manner.

Ethylene is known to promote internode elongation in deep-water rice plants (Minami et al., 2018). In cotton, ethylene is the major phytohormone that stimulates fiber cell elongation (Shi et al., 2006). While, the role of ethylene in the internode development is not yet completely understood. Previous studies showed that anti-GA growth retardants, such as PAC and uniconazole, suppress ethylene synthesis by blocking ACO (Kraus et al., 1992; Min and Bartholomew, 1996). In this study, not only ACO, but also SAM synthase and ACS were significantly suppressed by MC (**Figures 5D** and **8**). *ETR2* (ethylene receptor) and *EBF1* (ethylene signaling) were inhibited by MC, too. Zhang et al. (2015) reported that PAC repressed the expression of ethylene signal transduction genes in the dwarfing scape of *Agapanthus praecox*. Exogenous GA_3_ increased the expression of *ETR*, but did not affect *ACOH* (**Figure 8**), consistent with a previous report that the expression of ethylene-related genes were altered differently by exogenous GA (Di et al., 2019). Similar expression levels of *ACOH* and *ETR* between MC+GA_3_ treatment and control may result from the antagonism of MC and GA, suggesting that MC modulates the expression of ethylene-related genes through inhibiting GA biosynthesis.

ABA is a negative regulator of stem elongation during submergence (Hirayama and Shinozaki, 2007). ABA content was reduced in response to submergence in the elongating internodes of rice (Minami et al., 2018). In our study, MC treatment increased the expression level of *NCED3*, which encodes a rate-limiting enzyme in ABA biosynthesis, and suppressed the expression of two *ABAH4/CYP707A4*, which encode ABA inactivation enzymes (**Figures 5E** and **8**). In addition, *ABI5*, an important positive regulator of ABA signaling, was activated, while PP2Cs and RAV1, negative regulators of ABA signaling, were suppressed. These results imply that ABA biosynthesis and signal transduction are were enhanced by MC. Induced ABA biosynthesis was also found in potato by CCC (Wang and Xiao, 2009), in mango by PAC (Upreti et al., 2013) and in duckweed by uniconazole (Liu et al., 2015). We also found that exogenous GA_3_ can repress the expression of *NCED3* and *ABI5* (**Figure 8**), which is in agreement with previous findings that exogenous GA repressed the expression of *NCED3* and key ABA-signaling genes (Liu et al., 2018; Zhang et al., 2019). MC-induced expression of ABA-related genes seems to be dependent on GA based on similar expression levels of these genes between MC+GA_3_ treatment and control.

### The Inhibition of Internode Elongation by MC Requires Many TFs

Several TF families, including bHLH, AP2-EREBP, Orphans, MYB, GRF, and TCP, have been characterized for their regulatory roles in plant growth; they are mainly related to the triggering of downstream signaling cascades of hormones and metabolites. *BEEs* or *PREs* are involved in phytochrome signal transduction. In Arabidopsis, the overexpression of *PRE1*, *PRE5*, or *PRE6* resulted in elongated hypocotyl compared with that in the wild-type seedlings (Lee et al., 2006). In this study, the reduced expression of five *BEEs* and six *PREs* by MC might limit cell elongation of cotton internodes, which could be attributed to the reduced GA, BR, and auxin levels (**Figure S6**).

GRFs, plant-specific TFs, are involved in the regulation of leaf development, stem elongation, root growth, fruit enlargement, and seed formation (Kim and Tsukaya, 2015; Omidbakhshfard et al., 2015). GRFs increased leaf size by enhancing cell number or cell size (Nelissen et al., 2015; Jiang et al., 2018). Loss-of-function mutants of Arabidopsis *AtGRFs* and dominant-repressed transgenic poplar plants exhibited small leaves (Kim et al., 2003; Jiang et al., 2018).The repression of *OsGRFs* resulted in dwarf rice plants with short internodes (Kuijt et al., 2014). Li et al. (2018) reported that the expression of *GRFs* was reduced by PAC. In this study, the repressed expression of twelve *GRFs* in cotton by MC suggested the important roles of *GRFs* in MC-induced inhibition of internode elongation (**Figure S6**).

The TCP family has been subdivided into classes I and II in Arabidopsis. Class I TCPs are thought to promote growth, whereas class II genes inhibit growth (Li et al., 2005). However, this classification is not always clear. In Arabidopsis, cell proliferation is promoted in young internodes, but inhibited in leaves by AtTCP14, a member of class I TCPs (Kieffer et al., 2011). In contrast, gain-of-function and loss-of-function mutants of class II TCP lead to increased and decreased hypocotyl cell length, respectively (Challa et al., 2016). In the present study, the reduced expression of *TCP14*, *TCP7* (class I TCP), and *TCP4*, *TCP5* (class II TCP) in MC-treated internodes might contribute to the inhibition of internode elongation. Davière et al. (2014) showed a positive function of class I TCP factors in GA-mediated control of plant height. Therefore, the suppression of *TCP14* and *TCP7* by MC may be related to the reduced GA (**Figure S6**).

ANT and AIL, AP2-type TFs, are positive regulators of cell proliferation and organ growth in plants. ANT has been suggested to promote plant growth by activating the expression of *CYCD3* (Mizukami and Fischer, 2000). In Arabidopsis, overexpression of *ANT* increased leaves size, while *ant* mutants show reduced size of leaves (Mizukami and Fischer, 2000). Arabidopsis *ant ail6* double mutants exhibited reduced stature and smaller rosette leaves because of the reduced cell expansion or cell number (Krizek et al., 2016). BBX proteins are also positive regulators during plant growth and development such as seedling photomorphogenesis and shade avoidance (Holtan et al., 2011; Preuss et al., 2012; Crocco et al., 2015; Wang et al., 2015a). In Arabidopsis, the inhibition of *BBX19* or *BBX24* expression repressed hypocotyl elongation (Crocco et al., 2015; Wang et al., 2015a); overexpression of *BBX32* promoted hypocotyl growth (Holtan et al., 2011). In this study, MC inhibited the expression of two *ANTs*, one *AIL6*, six *BBX19*, two *BBX24*, and six *BBX32* in cotton internode (**Figure S6**), suggesting these TFs were involved in MC-induced inhibition of internode elongation.

### Lignin and Flavonoid Biosynthesis Was Inhibited by MC Treatment

Lignin is a kind of major structural component of the secondary cell wall; plants defective in lignin biosynthesis usually show severe growth retardation. The loss of function of CSE—catalyzing caffeoyl shikimate to caffeic acid—in *Arabidopsis* and *Medicago truncatula* results in reduced lignin levels and severe dwarfing (Vanholme et al., 2013; Ha et al., 2016). The transgenics of *alfalfa* and *Arabidopsis* with downregulated *HCT*, which catalyzes the conversion of *p*-coumaroyl-CoA to *p*-coumaroyl shikimate, are severely dwarf (Hoffmann et al., 2004; Shadle et al., 2007). LAC and PRX are oxidative enzymes that activate the monomers for combinatorial coupling into lignin in the cell wall (Meents et al., 2018). Triple *lac4*/*lac17*/*lac11* mutants cause dwarf Arabidopsis plants with little lignin (Zhao et al., 2013). In our study, MC application significantly reduced expression of most of the lignin biosynthetic genes, including *CSE*, *4CL*, *HCT*, *CCR2*, *CAD*, *LAC*, and *PRX* (**Figure S5A**), suggesting that lignin biosynthesis was inhibited by MC. The GA content was found to be positively correlated with lignin formation in plants (Biemelt et al., 2004). Björklund et al. (2007) reported that lignin biosynthesis genes induced by GA were also strongly induced by IAA in *Populus*. The decreased GA and auxin contents in MC-treated internodes may inhibit lignin biosynthesis, thereby causing the dwarf phenotype of cotton seedlings.

Flavonoids have been documented to affect plant growth and development mainly by negatively regulating auxin transport (Brown et al., 2001; Dare et al., 2013). Flavonoid metabolism has been reported to remain active in the early fiber cell elongation stage of cotton (Tan et al., 2013). The important roles of flavonoid in this process were mainly discovered in plants with the reduced expression of *CHS* (Brown et al., 2001; Dare et al., 2013). Arabidopsis *CHS* mutants (*tt4*) and *CHS*-silenced apple plants showed reduced plant height and increased rates of auxin transport (Brown et al., 2001; Dare et al., 2013). Our RNA-Seq data showed that the expression of two *CHSs* was significantly reduced (even by more than 2-fold) 48 and 72 h after MC treatment (**Figure S5B**). In addition, other flavonoid genes such as *LDOX*, *FLS*, and *F3H* were significantly downregulated in response to MC (**Figure S5B**). Therefore, flavonoid metabolism may be involved in the internode elongation of cotton and can be inhibited by MC treatment.

In summary, we conducted the histological, transcriptomic, and phytohormone analyses to better understand the mechanisms by which MC inhibits cotton internode elongation. We found that MC reduced internode length by inhibiting not only cell elongation but also cell division. MC altered the biosynthesis and signaling transduction of not only GA but also other plant hormones including auxin, BR, CK, ethylene, and ABA. We further proved that MC affected other plant hormones in a GA-dependent manner. In addition to hormones, many growth TFs, secondary metabolism associated genes were also remarkably altered by MC. This study was performed mainly at the transcription level, further studies, for example, at the protein level, need be conducted to identify the functions of the identified genes.

## Data Availability Statement

The sequencing data are deposited in NCBI Sequence Read Archive (SRA, http://www.ncbi.nlm.nih.gov/Traces/sra) with accession number PRJNA555146.

## Author Contributions

Y-FL and LW conceived the idea and supervised the research. LW, YY, and L-FW grew cotton seedlings and performed MC treatment, samples collection and RNA extractions. YY and L-FW performed histological analysis. LW, YY, L-FW MW, MZ, and YT performed DEG data analysis. YY and L-FW performed qRT-PCR analyses. LW and Y-FL wrote the manuscript. All authors read and approved the final manuscript.

## Funding

This work was supported by the National Natural Science Foundation of China (Grant no. 31601241 and 31771703), and the Research Fund of the major program of Science and Technology in Henan Provincial Committee of Education (Grant no. 17A180007). The funding organizations provided the financial support to the research projects, but didn't involve in study design, data collection, analysis, and manuscript writing.

## Conflict of Interest

The authors declare that the research was conducted in the absence of any commercial or financial relationships that could be construed as a potential conflict of interest.
