# Supplementary figures and images for: Transcriptome Profiling of the Elongating Internode of Cotton (Gossypium hirsutum L.) Seedlings in Response to Mepiquat Chloride
Source: Front Plant Sci. 2020 Jan 28;10:1751. doi: 10.3389/fpls.2019.01751 (PMC6997534; doi:10.3389/fpls.2019.01751)

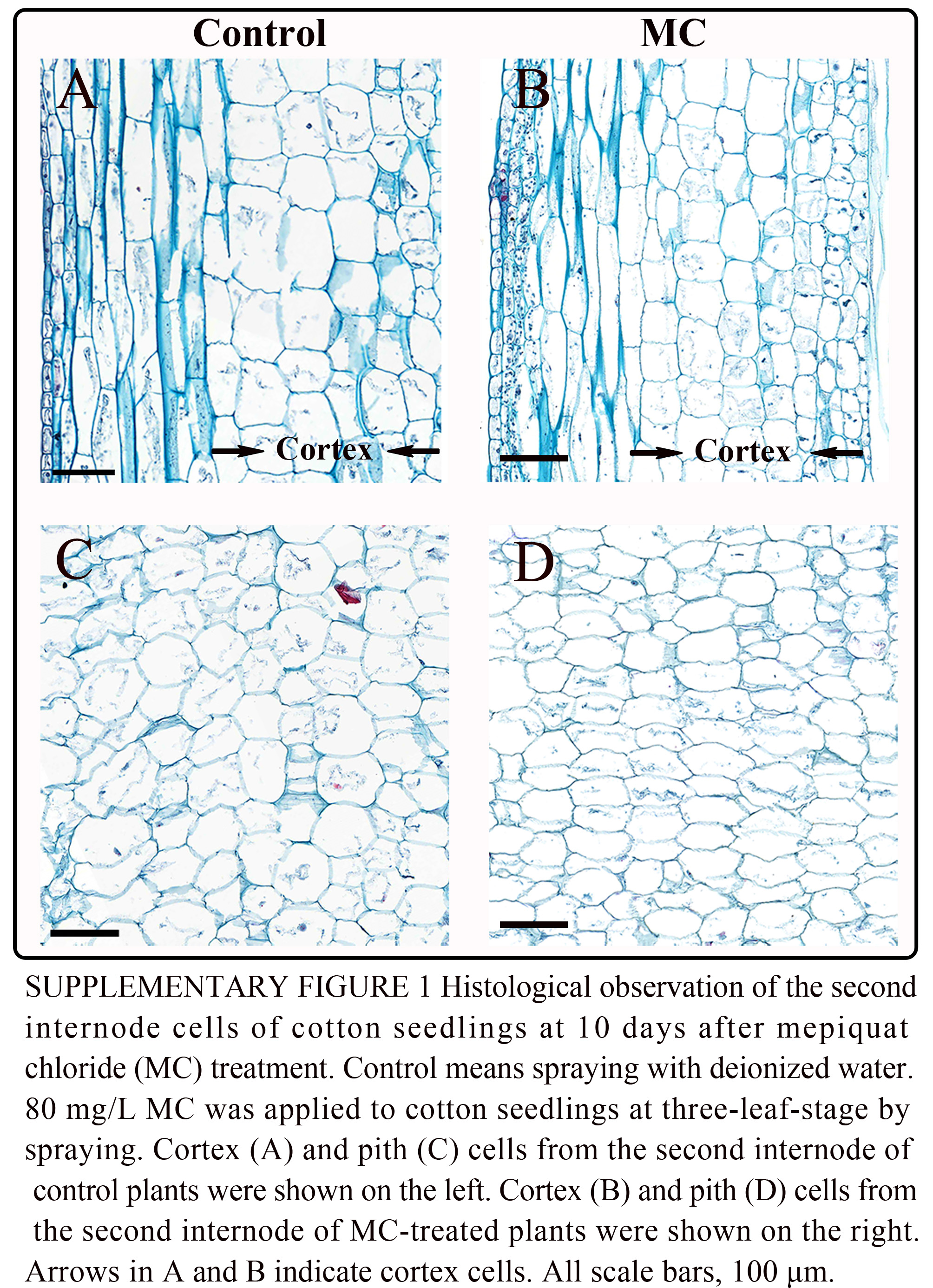

Supplement: Supplementary file 1 [file Image_1.jpg]

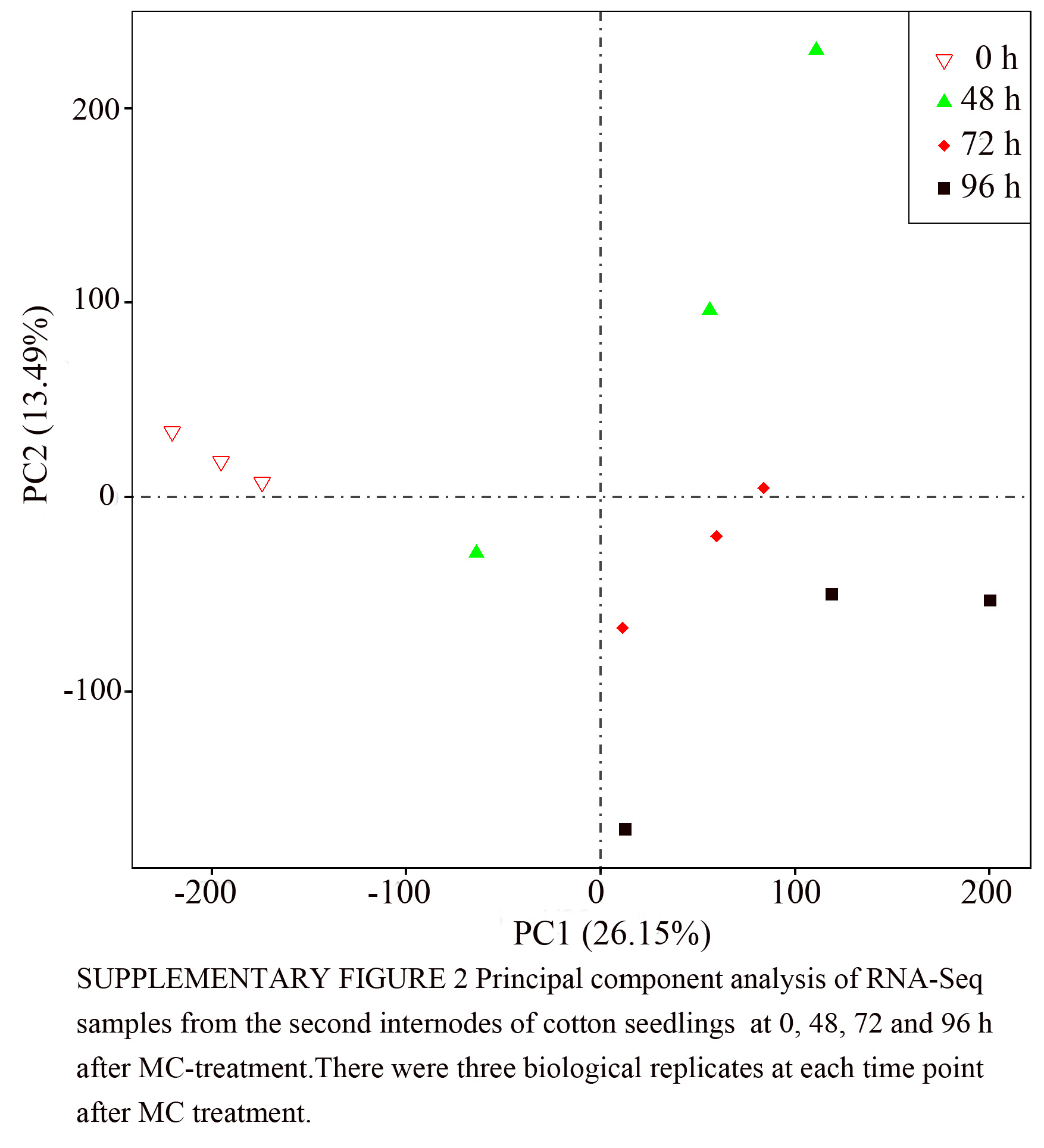

Supplement: Supplementary file 2 [file Image_2.jpeg]

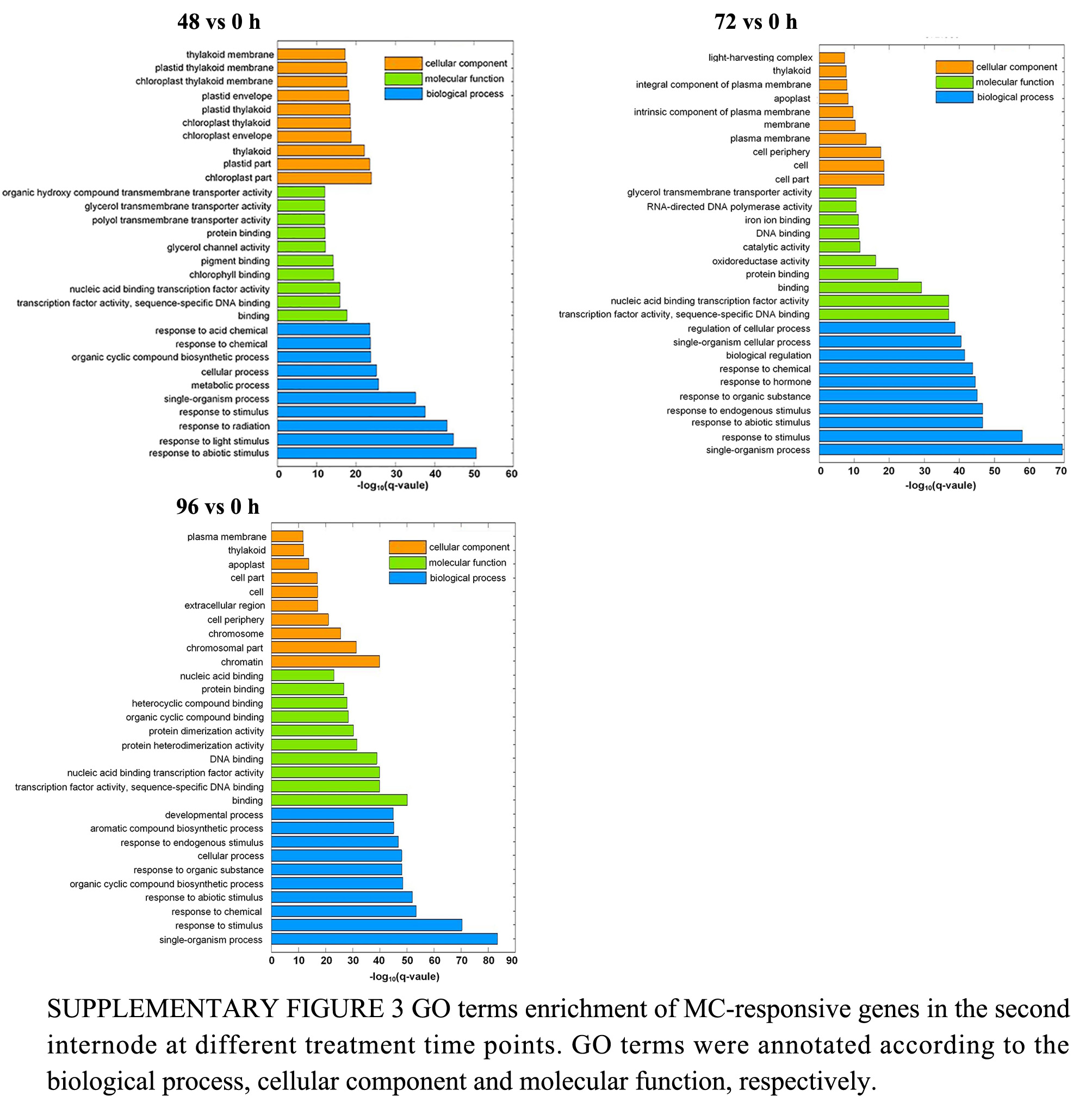

Supplement: Supplementary file 3 [file Image_3.jpeg]

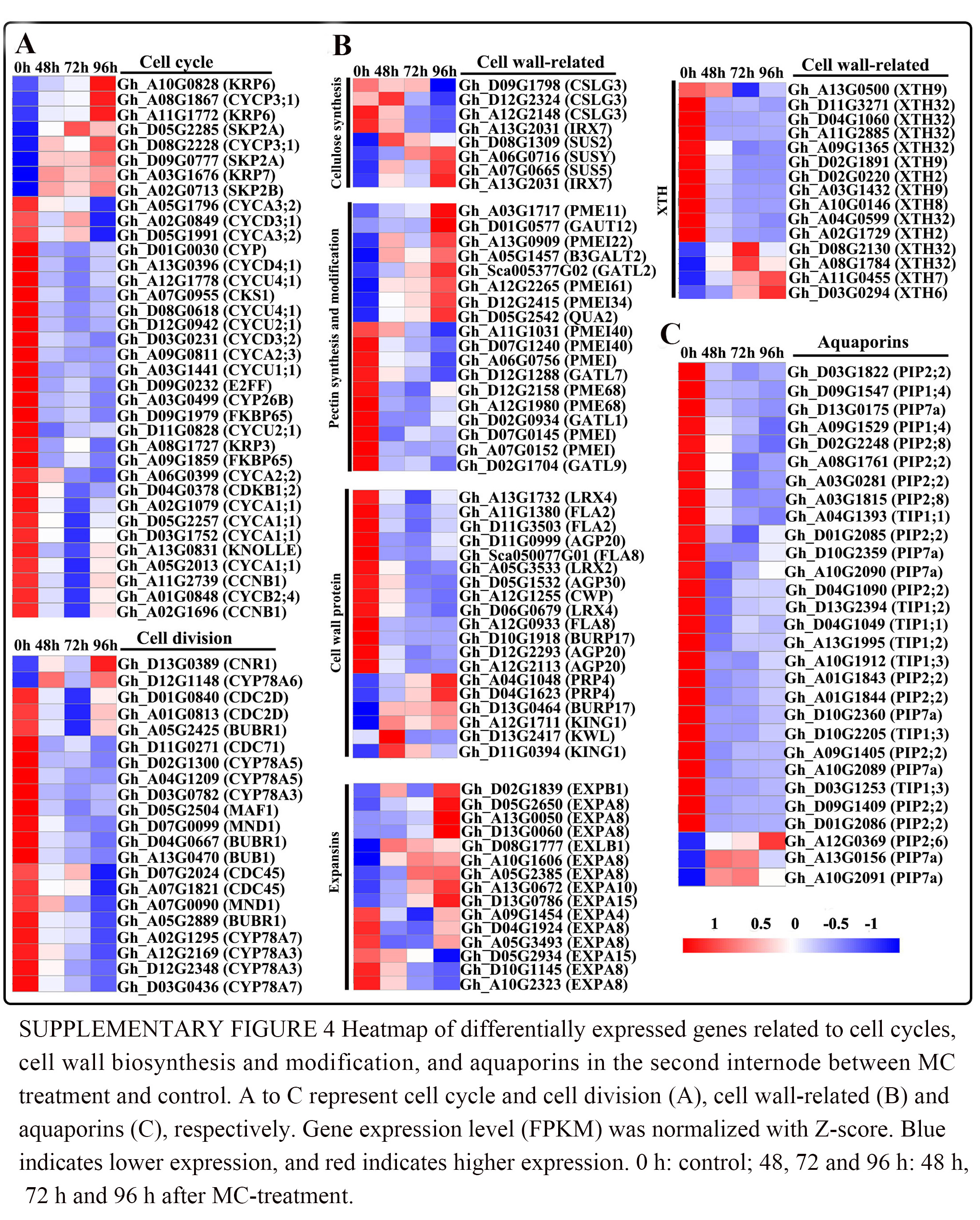

Supplement: Supplementary file 4 [file Image_4.jpg]

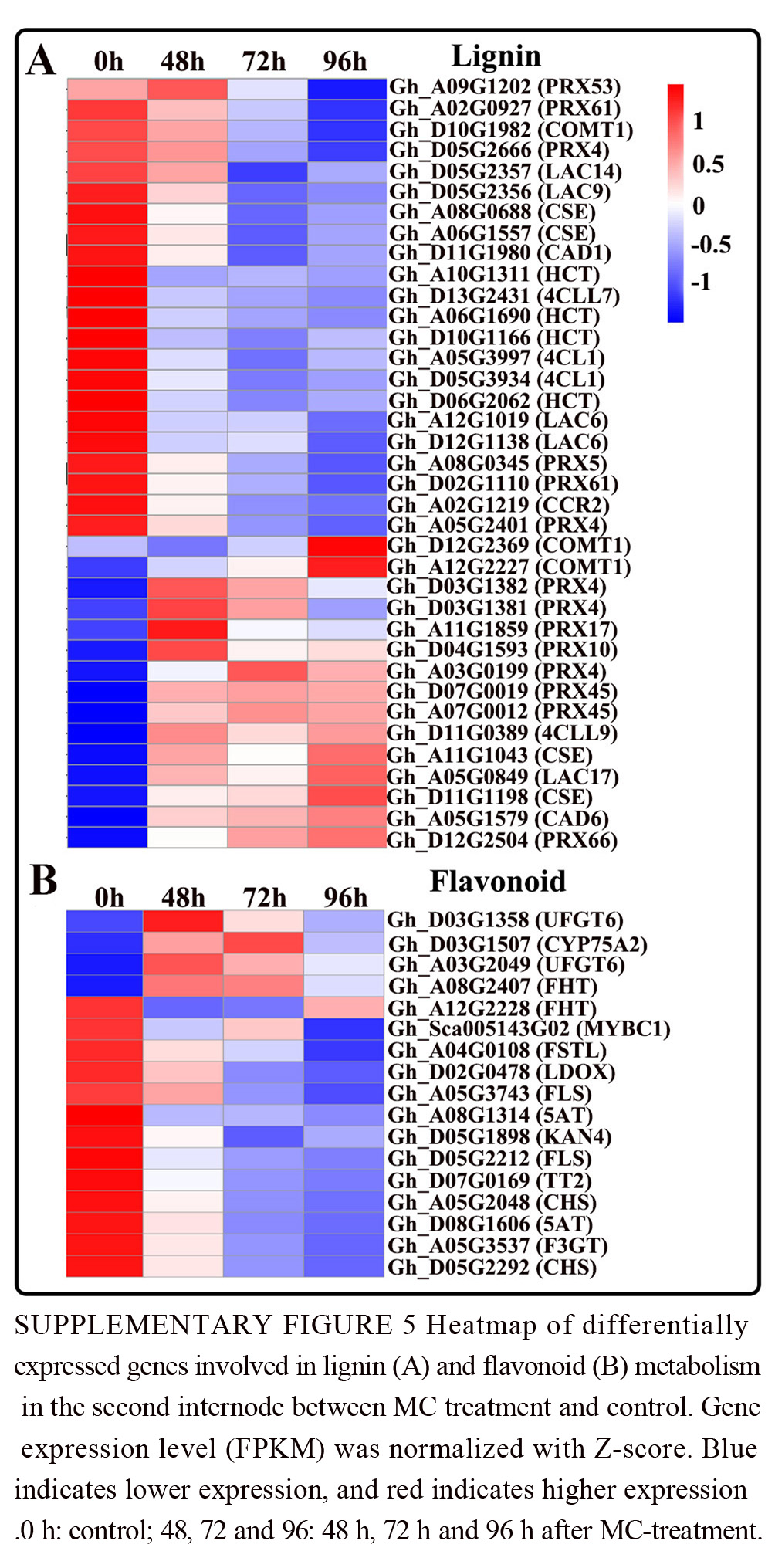

Supplement: Supplementary file 5 [file Image_5.jpg]

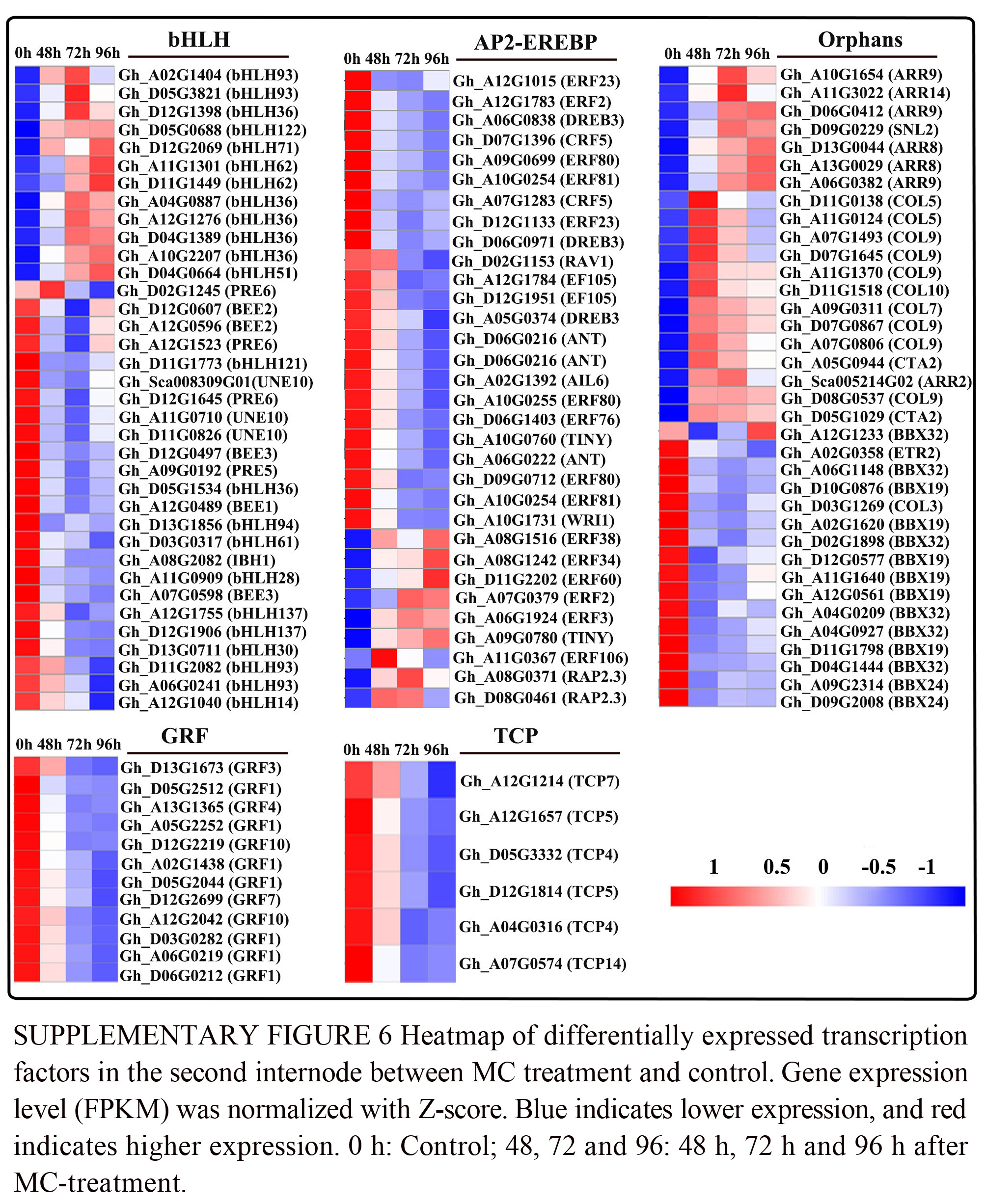

Supplement: Supplementary file 6 [file Image_6.jpeg]
