# Supplementary material for: Transcriptome Profiling of the Elongating Internode of Cotton (Gossypium hirsutum L.) Seedlings in Response to Mepiquat Chloride
Source: Front Plant Sci. 2020 Jan 28;10:1751. doi: 10.3389/fpls.2019.01751 (PMC6997534; doi:10.3389/fpls.2019.01751)
Supplement: Supplementary file 7 [file Table_1.doc]

| Gene ID | Gene name | Forward primers (5'-3') | Reverse primers (5'-3') |
| --- | --- | --- | --- |
| Gh_D04G0598 | *Actin4* | AACGTTTCAGATGCCCCGAA | TCCTTGCTCATTCTGTCGGC |
| Gh_D12G0497 | *BEE3* | TCTCCTTCACTGTGCTTGCC | GCTTGCCCTCTTCTTGCTCT |
| Gh_D06G0212 | *GRF1* | TGGGGAGCTTTTCGTCTTGG | GGCCTGATTGACCTTCCACA |
| Gh_D03G0231 | *CCD3* | GAGCCCGAGTAGTCCTAATGG | ACCAGATCGACAAACATACGGT |
| Gh_D02G1300 | *CYP78A5* | TGGGCTCTTCTCTTGGTTGG | ACTTCACAGCGTTGAGGGTT |
| Gh_D11G3261 | *WOX8* | GCACAAATCCATGTCTGCCC | TTCTGCTTGCTTGGTGTCCC |
| Gh_A07G1786 | *IBH1* | CCACAGAGATCTTCCGACGG | CCCAACCCCATCTTCCCTTC |
| Gh_D13G2273 | *SCL3* | CGCCCTTATCTTCACCCGTT | CCCTTGAGACCGTGGATTCC |
| Gh_D07G0786 | *RALFL27* | CAAGTGGTGTTGTTCGGTGA | ACTCCCCTCTTGCAACGATT |
| Gh_D01G1218 | *TBR* | CGGGCATTGGTGGAATCATG | GTCAGTGCTCTTCGAAACGC |
| Gh_A01G0386 | *LSH10* | TCGCAGAAGAGACGGGATTG | AAGAGGGCACGTACATGGTC |
| Gh_A05G0290 | *CKX7* | CGGGATGCTGAGTTTCTGGT | CCACTTCGAGGCAGTACAGG |
| Gh_A07G1951 | *SCR* | TGGCCGGCTTTGTTCCATAT | CGTGATGTCGATTTCCCCGA |
| Gh_D02G0734 | *PIN6* | GAGCAGTGTTCGGGGATCAA | AAGTGGCGAGGGAGAAAAGG |
| Gh_D08G0763 | *TIR1* | CCTGACTCTCGAGCCACTTG | ACCCGGAAAGCACATGATGT |
| Gh_D01G0557 | *GH3* | CACGCCCTGTTCTCACTAGC | AGGACCTCTTTGTGTTGGCA |
| Gh_D09G1630 | *TAR2* | GGCGACGAAGACAGGGTTAT | TCTCCGTCACTGCATTTCCC |
| Gh_D01G0840 | *CDC2D* | ATACATCCGCTCCTTTCGCC | GCTAGGCCGAGATCAGCTAT |
| Gh_A02G1079 | *CCA1* | GCTCCATCACTTATCGCTGC | TTTCTCCCTGATTGCTGGCA |
| Gh_D04G0378 | *CKB1* | CGTCGACACCAACAAGAACG | TTGAGATCGCGATGGAGGAC |
| Novel06146 | unannotated | GGTGGAAGAGATAGGTGCGG | CAATGGTAGCTGACTGGTAATGC |
| Gh_D01G0300 | *GA2OX1* | AGCCTGTGAAGAGTTTGGGT | TCACCGTGCATACCAATCCT |
| Gh_D06G0560 | *DELLA* | GTCGACTCGCTGCTAACTGA | TCTTCTTCCATGGCCGTGAC |
| Gh_A11G3078 | *IPT3* | CCTGGATTCGACCGCTACTT | AATTCCGCCACTATCTCCGT |
| Gh_A12G0902 | *PUP11* | CAAGCTGCCTATCGTCTACC | TCAACCCCACAACACCAACA |
| Gh_A10G2109 | *CPD* | TCGCACGTTCAATCCATGGA | AGCTTATCTTCTTCGGCCGG |
| Gh_A08G2060 | *NCED3* | ACCCACTTCACGAACCAGTC | CATGGAGTTCACCGATGGCT |
| Gh_A01G1768 | *ABI5* | CGCCGCATTCACAACATCAA | CCACCAGCTGCACCTACTTT |
| Gh_A09G1977 | *ACOH* | GGCATCAAAGGACTCGTCGA | GCAGGCGTTACGGACTTCAT |
| Gh_A02G0358 | *ETR* | TGCTGTGCGGATTGACTCAT | TGCAAACCGGTCTCCTTCTG |

Table S1 Primers used for qRT-PCR assays in this study
